# A genome-wide identification, characterization and functional analysis of salt-related long non-coding RNAs in non-model plant *Pistacia vera* L. using transcriptome high throughput sequencing

**DOI:** 10.1038/s41598-020-62108-6

**Published:** 2020-03-27

**Authors:** Masoomeh Jannesar, Seyed Mahdi Seyedi, Maryam Moazzam Jazi, Vahid Niknam, Hassan Ebrahimzadeh, Christopher Botanga

**Affiliations:** 1 Department of Plant Biology, School of Biology, College of Science, University of Tehran, Tehran, Iran; 2 Plant Biotechnology Department, National Institute of Genetic Engineering and Biotechnology, Tehran, Iran; 3 Research Institute for Endocrine Science (RIES), Shahid Beheshti University of Medical Sciences, Tehran, Iran; 4 Department of Biological Sciences, Chicago State University, Chicago, Illinois United States of America

**Keywords:** Bioinformatics, Biological techniques, Molecular biology, Physiology, Plant sciences

## Abstract

Long non-coding RNAs (lncRNAs) play crucial roles in regulating gene expression in response to plant stresses. Given the importance regulatory roles of lncRNAs, providing methods for predicting the function of these molecules, especially in non-model plants, is strongly demanded by researchers. Here, we constructed a reference sequence for lncRNAs in *P. vera* (*Pistacia vera* L.) with 53220 transcripts. In total, we identified 1909 and 2802 salt responsive lncRNAs in Ghazvini, a salt tolerant cultivar, after 6 and 24 h salt treatment, respectively and 1820 lncRNAs in Sarakhs, a salt sensitive cultivar, after 6 h salt treatment. Functional analysis of these lncRNAs by several hybrid methods, revealed that salt responsive NAT-related lncRNAs associated with transcription factors, CERK1, LEA, Laccase genes and several genes involved in the hormone signaling pathways. Moreover, gene ontology (GO) enrichment analysis of salt responsive target genes related to top five selected lncRNAs showed their involvement in the regulation of ATPase, cation transporter, kinase and UDP-glycosyltransferases genes. Quantitative real-time PCR (qRT-PCR) experiment results of lncRNAs, pre-miRNAs and mature miRNAs were in accordance with our RNA-seq analysis. In the present study, a comparative analysis of differentially expressed lncRNAs and microRNA precursors between salt tolerant and sensitive pistachio cultivars provides valuable knowledge on gene expression regulation under salt stress condition.

## Introduction

Pistacia belongs to Anacardiaceae family and contains 13 or more species, which among them pistachio *(Pistacia vera* L.) is the only cultivated and economically important species that is called as the ‘green gold tree’. The other species are used mainly as a rootstock for pistachio cultivation^1,2^. Like many other fruit trees, *P. vera* is hard to root and thus requires a rootstock for vegetative propagation^3^. Pistachio is a deciduous (2n = 30), wind-pollinated tree species^4,5^. Studies indicate that pistachio probably originated in the Middle East^6–8^. It has a long history of cultivation (3000–4000 years) in Iran^9^. In 2017, Iran has been ranked first in area harvested (429535 ha) and production quantity (574987 tons) of pistachio among United States, Turkey, and Syria as biggest pistachio producer countries^10^. According to the number of pistachio genotypes, Iran is one of the rich countries in the world^7,8,11^. In addition to fruit, other parts of plant, including leaf, flower and resins, have pharmacological properties^12,13^.

Salinity is major and ever-increasing problem in arid and semi-arid regions that affect plant production and growth throughout the world^14^. The cultivated areas of pistachio in Iran are often in arid and semi-arid regions that the most important problem for economic crop production in these areas is high concentration of ions specially NaCl^15^. Although *P. vera* is known as a salt tolerant species, but, its yield is significantly affected by high salinity conditions ^16–18^. Consequently, study of salt tolerance mechanisms and identification and introduction of salt tolerant pistachio rootstocks have a specific importance to establishment of new pistachio orchards and also forestation in arid and salinized zones^15^. However, few studies have been conducted at the genetic and molecular levels on this commercial crop. Researches on pistachio are often focused on genetic diversity, genetic relationships and sex determination by using of different molecular markers such as simple sequence repeat (SSR)^19–21^, amplified fragment length polymorphism (AFLP)^22,23^, inter-simple sequence repeats (ISSR)^5,24–27^, randomly amplified polymorphic DNA (RAPD)^28–30^, retrotransposon microsatellite amplified polymorphism (REMAP)^31^, selective amplification of microsatellite polymorphic loci (SAMPL)^32^ and single nucleotide polymorphism (SNP)^33^. It has been reported the genome survey of *P. vera* cv. Siirt and for the first one the data on the structure of the pistachio genome was published. Their results provided the 26.77 Gb assembly data that used for the discovery of simple sequence repeats (SSR), which 59,280 SSR motifs were obtained and 206 SSR primers were applied to characterize of 24 *P. vera* cultivars and 20 wild Pistacia genotypes. They indicated that the pistachio genome is about 600 Mb in size and is highly heterozygous^34^. In addition to genomic studies, in our previous work, whole transcriptome sequencing was done using Illumina Hiseq 2000 platform by pooling sample representing 24 different tissues and treatments of two pistachio cultivars for construction of pistachio reference transcriptome. All obtained contigs of pistachio transcriptome were functionally annotated^35^. This study was the first large scale report on transcriptomic research and gene discovery in this species. Moreover, genetic diversity, domestication, and structure of pistachio populations have been studied using the genome and transcriptome analysis of 107 cultivars and wild *P. vera*, and also 35 related wild Pistacia. Comparative genomic analysis showed that stress adaptation of pistachio is probably related to the expanded cytochrome P450 and chitinase gene families^36^.

Non-coding RNAs (ncRNAs) are functional and low protein-coding potential RNA molecules longer than 200 nt, which can be classified in different ways according to their function, location and length. According to their length, ncRNAs can be divided into small ncRNAs (sRNAs) (20–30 nt) that commonly found as transcriptional and translational regulators, and also medium-sized ncRNAs (50–200 nt) and long ncRNAs (lncRNAs) (>200 nt) that usually involved in other processes, such as splicing, gene inactivation, and translational regulation^37^. To date, the best-studied ncRNAs in plants are sRNAs include microRNAs (miRNAs), small interfering RNAs (siRNAs) and natural antisense siRNAs (nat-siRNAs), which play important role in determining the comp of genes in the transcriptome through transcriptional and post-transcriptional regulation of gene expression^38,39^. Initially, lncRNAs were thought of transcriptional noise which could have been due to their low levels of sequence conservation and expression compared with mRNAs^40^. However, accumulating evidence showed that lncRNAs play important roles in biological processes of organisms^41^. Further studies indicated that they extensively distribute in different part of the genomes including introns, intergenic regions, antisense transcripts, pseudogenes and retrotransposons^42^. They involve in gene expression regulation in different ways such as interaction with DNA, mRNA, miRNA and protein that consequently lead to regulation of gene expression at the transcriptional, post-transcriptional, epigenetic, translational, and post-translational levels. They are involved in the regulation of histone modifications at the chromatin level, DNA methylation at the DNA level, and transcriptional activation and interference. In post-transcriptional regulation, they act as a precursor of miRNAs and siRNAs and miRNA sponges, regulate alternative splicing, mediate RNA decay, regulate RNA stability, involve in protein relocalization and regulate RNA methylation of mRNA. lncRNAs are involved in translational regulation and post-translational modification. These molecules regulate protein phosphorylation, ubiquitination, and acetylation^43^. lncRNAs can act as a gene expression regulators during developmental stages of tissue and in response to external stimuli^44^. Recently, a great number of lncRNAs had been identified in animals and humans but only a few of them have been functionally annotated^45–48^. Extensive studies have been done on lncRNAs in humans and animals, but such works in plants are relatively rare, and only limit to some model plants^49–51^. The advent of high-throughput sequencing technologies have facilitated the identification and characterization of many lncRNAs in plants, such as *Arabidopsis thaliana*^52,53^, *Medicago truncatula*^54^, *Triticum aestivum*^55^, *Oryza sativa*^56^, *Solanum lycopersicum*^39^ and *Zea mays*^57^. There is an increasing number of evidence showing that plant lncRNAs have key roles in genomic imprinting, cell differentiation, epigenetic regulation, and stress responses^51,58,59^. To introduce stress responsive lncRNAs in plant, identification of differentially expressed lncRNAs during abiotic and biotic stress conditions can be very helpful. To date, lncRNAs responsive to powdery mildew infection, heat and drought stress in wheat^55,60^, drought, salt and cold stress in Arabidopsis^61^, salt stress in rice^62^ TYLCV infection in tomato^63^, osmotic and salt stress in *Medicago truncatula*^64^ and drought and salt stress in Maize^49,65^ have been reported.

Although identifying lncRNAs with the advent of next-generation sequencing technologies have been expanded in plants but the characterization and annotation of these regulatory molecules have not been expanded and still in its infancy. Thus, the use of new bioinformatics methods for functional prediction of lncRNAs, especially in plants without reference genome which often have been encountered various challenges in the characterization of lncRNAs, can be helpful to increase our knowledge about the regulatory pattern of these molecules. The objectives of this study were, (1) create a comprehensive reference sequence assembly for lncRNAs from leaf, stem, and root under control and salinity conditions in *P. vera*, (2) use new methods for functionally analysis of lncRNAs, (3) identify of miRNA precursors in *P. vera*, (4) study of the biological pathways in putative target genes of salt responsive lncRNAs and miRNAs, (5) develop novel SSR markers from lncRNA sequences for application in Pistacia species, and (6) analyze selected salt responsive lncRNAs, pre-miRNAs and mature miRNAs using qRT-PCR.

In this study, we used RNA-seq data for constructing a comprehensive reference (53220 sequences) for lncRNAs in *P. vera*. Of these 53220 lncRNAs, 62 and 36 transcripts were found to be homologous to existing miRNA precursors and conserved lncRNAs, respectively. Moreover, we performed transcriptional gene expression profiling to identify salt responsive lncRNAs in pistachio plant. We filtered and annotated of salt responsive transcripts and then experimentally validated them using reverse transcription-quantitative PCR. According to our expectations, qRT-PCR results confirmed that all selected salt responsive lncRNAs respond to salt stress. These results suggested that at least a subset of these newly identified differentially expressed lncRNAs may play important roles in response to salinity stress in this plant. These results extend the current knowledge on lncRNAs as important gene expression regulators under stress conditions. The present study is the first report on the lncRNAs identifying and profiling under salt stress in salt tolerance and sensitive cultivars of *P. vera*. The annotation methods adopted in this study can be used in the future for other plants without a reference genome. In addition, the results of this study will provide essential information for further studies such as functional and comparative genomic studies in pistachio. Also, new generation of lncRNA-SSR markers can be used for different marker based studies in this economically important species.

## Results

### De novo assembly and identification of putative lncRNAs

**Figure 1:**
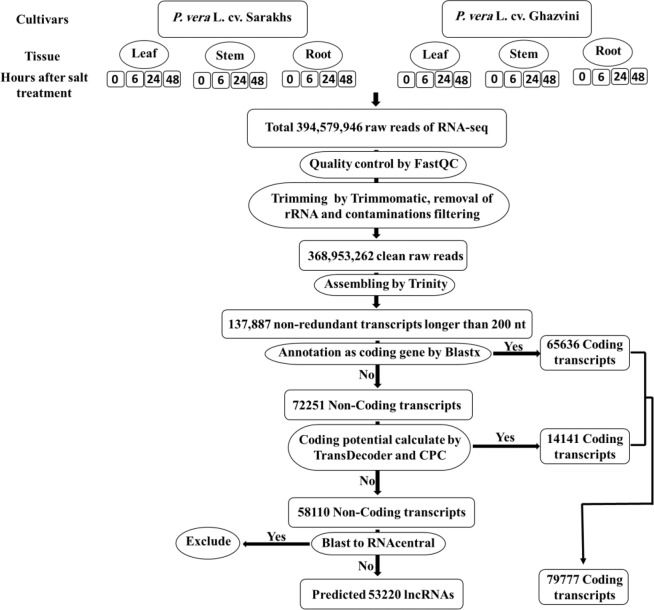
General flowchart of the pipeline used to identify of lncRNAs from reference transcriptome of *P. vera*.

In our previous study, RNA sequencing of pooled sample including three tissues (leaf, stem, and root) and two salt tolerant and sensitive pistachio cultivars under control and four time points after salt treatment was done by Illumina Hiseq 2000 platform for building a reference transcriptome^35^. RNA-seq produced 394,579,946 million raw reads as paired end (PE). After trimming of the low quality bases and adapters as well as the removal of rRNAs and contaminations, a total of 368,953,262 million clean PE reads were reminded for using as an input to our transcriptome assembling strategy, resulting in a total of 137887 non-redundant assembled transcripts in *P. vera* (Fig. 1).

**Figure 2:**
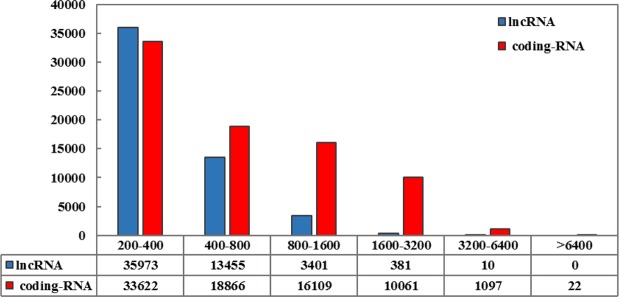
The distribution of sequence length in lncRNAs compared with protein coding genes.

To identify lncRNAs or to separate the non-coding and coding sections of transcriptome, we used Blastx first and then CPC and Transdecoder softwares were used to check the protein coding potential of contigs. Transcripts with the evidence of protein-coding potential were separated and classified as the coding section of transcriptome. All the remaining non coding transcripts after separating of miRNA precursor and conserve lncRNA sequences, were aligned against RNAcentral database to separate lncRNAs from other known small noncoding-RNAs. Finally, after adding miRNA precursor and conserve lncRNA sequences, we identified a total of 53220 long non-coding RNAs (Supplementary Table S1) that include 38.6% of total transcriptome sequences of *P. vera* (Fig. 1). This number of lncRNAs is bigger than the previously identified for maize, rice, tomato, kiwifruit, strawberry and Miscanthus^65–68^ and less than those in wheat^60^. The length of the lncRNAs ranged from 200 to 4716 bp. The majority (86%) of pistachio lncRNAs lengths were around 200–600 bp. This distribution of sequence length was similar to that of in Miscanthus^66^. The average and median length of lncRNAs was 404 bp and 317 bp, respectively. Thus, the pistachio lncRNAs length was shorter than those in rice and Miscanthus^66,68^ and longer than those in Arabidopsis and maize^61,65^. The length of protein coding transcripts ranged from 200 to 8431 bp, the majority (92%) of which were around 200–2000 bp. The length of mRNAs was 808 bp in average and 488 bp in median. The average and median length of lncRNAs were shorter than coding-RNAs (Fig. 2). In addition, the average GC content for lncRNAs was 36% but this average in coding transcripts was detected as 40% that relatively higher than lncRNAs.

### Identification of conservation in lncRNAs

lncRNAs similar to protein-coding genes may have conserved sequences and subsequent similar functions in different plant species even though a lack of protein constraints may provide conditions for rapid changes in DNA sequences. In this study, to investigate the conservation of Pve_lncRNAs (*P. vera* lncRNAs) between different species, we aligned lncRNA sequences of *Arabidopsis lyrata*, *Citrus clementinawere*, *Citrus sinensis*, *Malus domestica*, *Populus trichocarpa* and *Zea mays* against the Pve_lncRNAs; 36 Pve_lncRNAs were found to share similarities with certain sequences in the respective plants (Supplementary Table S2). The number above suggest that about 0.07% of the Pve_lncRNAs have potential homologs in other species. Thus, the evolutionary conservation of Pve_lncRNAs is limited. Our results are similar to previous work which reported the primary sequences of lncRNAs have very little conservation compared to mRNAs, but the secondary and tertiary structures of these molecules have a high conservation. Such a pattern of sequence and structural conservations in lncRNAs might be related to their specific regulatory functions of these molecules^69,70^.

### Detection of SSRs and transposons in lncRNAs and coding-RNAs

Given the important role of SSRs as a molecular marker for genetics and biological researches and the key regulatory role of lncRNAs, maybe it’s time that to focus more on lncRNA-SSRs as a new generation of molecular markers with higher efficiency and specificity. All the 53220 and 79777 transcripts of lncRNAs and coding-RNAs, respectively, were used to find potential microsatellites by MISA. The SSR loci detection were done by searching of two to six nucleotides motifs with a minimum of 6,5,5,4 and 4 repeats, respectively. To this end, 2166 and 4148 microsatellites were detected in Pve_lncRNAs and coding-RNAs, respectively (Supplementary Table S3). The percentage of microsatellites containing transcripts with 1% difference in Pve_lncRNAs and coding-RNAs was 4% and 5%, respectively. Among the microsatellites, di-nucleotide motifs were the most abundant types in lncRNAs (57.6%) but the most abundant motif types in coding-RNAs was tri-nucleotide motifs (50.2%). Other motifs followed by di-nucleotide (43.2% in coding-RNAs), tri-nucleotide (34.2% in lncRNAs), tetra-nucleotide (1.9% in lncRNAs and 0.6% in coding-RNAs), penta-nucleotide (4.2% in lncRNAs and 3.5% in coding-RNAs) and hexa-nucleotide (2% in lncRNAs and 2.5% in coding-RNAs). The di-nucleotide repeat AG/CT was the most abundant motif detected in both lncRNAs (26%) and coding-RNAs (23.4%). The second most frequent motif was AT/AT in both lncRNAs (21.5%) and coding-RNAs (15.9%). The third most frequent motif was AC/GT (10%) in lncRNAs and AAG/CTT in coding-RNAs (15%) (Supplementary Fig. S1).

In order to find putative transposon sequences in lncRNAs and coding-RNAs, initial transposon filtering of Pve_lncRNAs and coding-RNAs was done by aligning these transcripts to transposon sequences of six plants including *Arabidopsis thaliana*, *Asparagus officinali*s, *Carica papaya*, *Morus notabili*s, *Populus trichocarpa* and *Phoenix dactylifera*. The 97 transcripts in Pve_lncRNAs and 1540 transcripts in coding-RNAs, was matched to transposon sequences of other plant and so were classified as probable transposon sequences. The percentage of transposon containing transcripts in lncRNAs was 0.2% but this percentage in coding-transcripts was detected as 1.9% that is about 10 times higher than the lncRNAs. In the next step, repeat-masking of probable transposon-lncRNA sequences against the repeat library of *Arabidopsis thaliana* indicate the 6 Retro elements and 1 DNA transposons.

### Expression pattern of lncRNAs under salt stress

**Figure 3:**
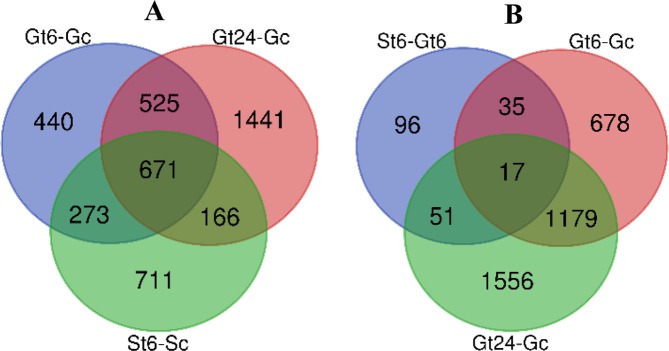
Common and specific salt responsive lncRNAs from two *P. vera* cultivars. (**A**) Venn diagrams show the number of common and specific differentially expressed lncRNAs between St6-Sc, Gt6-Gc and Gt24-Gc. (**B**) Venn diagrams show the number of common and specific differentially expressed lncRNAs between St6-Gt6 (differential expressed lncRNAs between Ghazvini and Sarakhs after 6 h salt treatment), Gt6-Gc and Gt24-Gc.

**Figure 4:**
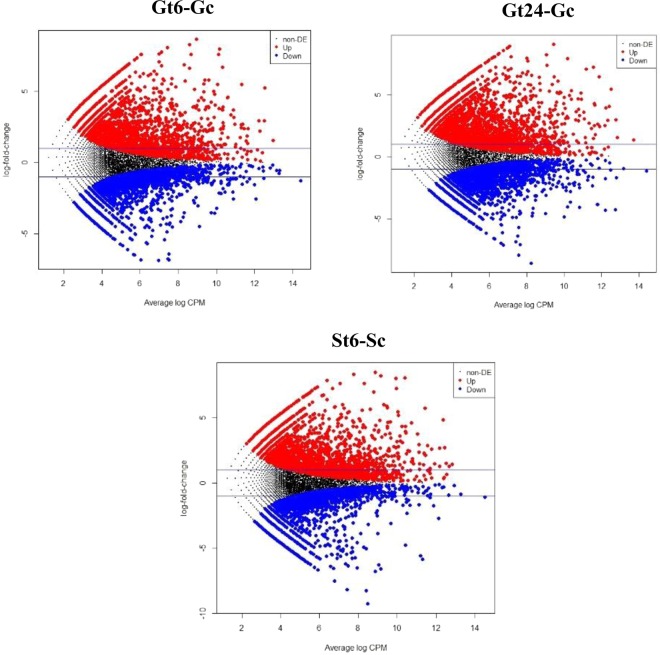
MD-plot showing the log-fold change of differential expressed lncRNAs compare to control and average abundance of each lncRNAs in three studied samples. Significantly up and down differential expressed lncRNAs are highlighted in red and blue, respectively. MD-plot was generated using EdgeR package^151^.

To investigate the role of lncRNAs in response to salt stress and also compare the expression pattern of lncRNAs in two salt tolerant and sensitive cultivars of pistachio, we analyzed the expression changes of these transcripts compared to control in root tissue of *P. vera* after 6 h (in Ghazvini and Sarakhs) and 24 h (in Ghazvini) of salt treatment. Differential expression analysis revealed a total of 1909 and 2802 stress responsive lncRNAs after 6 and 24 h of salt treatment, respectively, in Ghazvini, of which 440 and 1441 transcripts were exclusive in Gt6-Gc (differential expressed lncRNAs after 6 h salt treatment compares to control in Ghazvini) and Gt24-Gc (differential expressed lncRNAs after 24 h salt treatment compares to control in Ghazvini) differential expression sequences, respectively (Fig. 3A). Of a total of 1820 salt responsive lncRNAs, 711 transcripts were exclusively differentially expressed in Sarakhs. Of all the differential expressed lncRNAs, 671 non-coding transcripts were differential expressed in all cultivars and all conditions (Fig. 3A). MD-plots containing the log-fold change of differential expressed lncRNAs compare to control and average abundance of each lncRNAs in Gt6-Gc, Gt24-Gc and St6-Sc (differential expressed lncRNAs after 6 h salt treatment compare to control in Sarakhs) are shown in Fig. 4.

In this analysis, we introduced two different sets of lncRNAs that related to early and late responses to salt stress conditions. The differentially expressed lncRNAs under 6 and 24 h after salt treatment compared to control was defined as an early and late responses, respectively. In the early response, we identified 713 lncRNAs associated with this condition in Ghazvini. In a late response, we identified 1607 non-coding transcripts in Ghazvini. A total of 1196 lncRNAs were commonly expressed after 6 and 24 h of salt exposure compared to control, these transcripts were associated with both the early and late responses (Fig. 3A).

**Figure 5:**
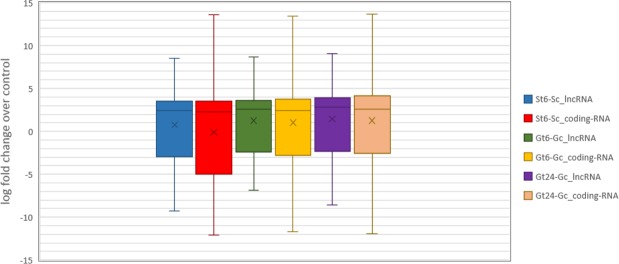
Expression pattern of lncRNAs and coding-RNAs in two *P. vera* cultivars in different conditions. The size of boxes represents the expression levels variability of the contigs.

It is reported that lncRNAs usually have lower expression levels compared to coding-RNAs^71,72^. To compare the expression level variability of lncRNAs and coding-RNAs, we draw box plot by log fold change of their transcripts over control in two studied salt tolerant and sensitive cultivars of *P. vera* under salt stress condition (Fig. 5). When comparing the boxes sizes of lncRNAs and coding-RNAs, the slightly lower expression variability of lncRNAs compared to coding-RNAs especially in the section of down-regulation is observable. The highest degree of expression variability difference between lncRNAs and coding-RNAs is in Sarakhs. As can be observed, Ghazvini-lncRNAs presented a similar variability in their expression levels compared to the control in almost all time points after salt treatment. Sarakhs-lncRNAs show slightly higher expression variability when compared to Ghazvini-lncRNAs but this difference is more in the coding section of *P. vera* transcriptome (Fig. 5).

### Functional analysis of lncRNAs

#### Blast based transcript filtering

**Figure 6:**
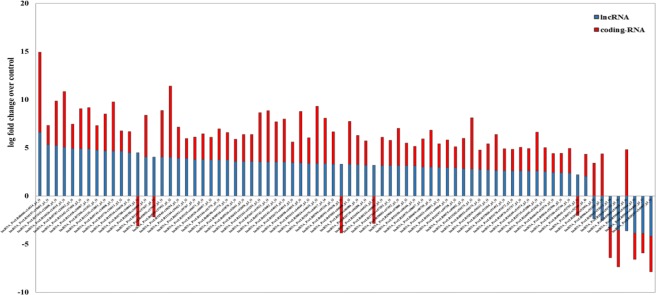
The log fold change value of 76 lncRNA-target pairs that were differentially expressed under salt stress predicted by Blast based transcript filtering and LncTar software.

**Figure 7:**
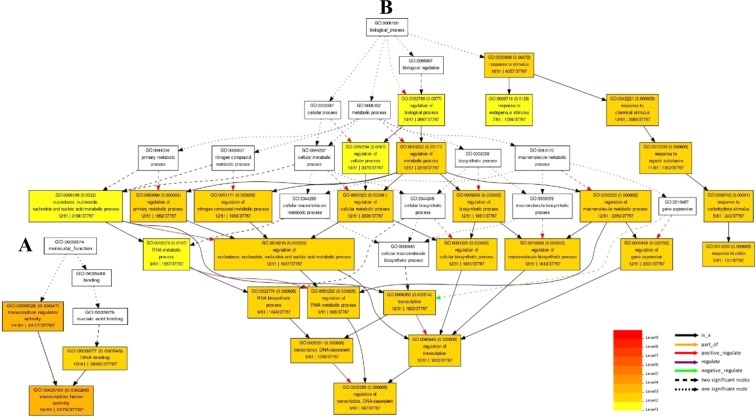
Potential functions of 76 lncRNA-target genes that differential expressed under salt stress predicted by Blast based transcript filtering method and LncTar software. (**A**) Graphical results of molecular function. (**B**) Graphical results of biological process. Graphical results were prepared through AgriGO web-based tool^153^.

For functional studies, the lncRNAs of Ghazvini as a salt tolerant cultivar was exclusively selected for analysis. In BLAST-based method, 3515 transcripts obtained from combination of all salt responsive lncRNAs of Gt6-Gc and Gt24-Gc, were aligned against salt responsive coding genes in Gt6-Gc and Gt24-Gc differential sequences. If the paired regions between two transcripts has a continuous matching region (without gap openings) longer than 150 nucleotides with ≥90% identity, they were classified as 150-nt lnc-coding RNA-NAT pairs. A total of 143 BLAST-predicted lncRNA-mRNA pairs was identified. These pairs were analyzed for lncRNA-mRNA hybridization by Lnc Tar software. The interaction of 76 lncRNA-mRNA pairs was confirmed by analyzing the results of this software (Supplementary Fig. S2). As can be observed in Fig. 6, 89.5% of lncRNA-NAT pairs showed a similar expression pattern, 82.9% up-regulation and 6.6% down-regulation. Only 10.5% of lncRNA-NAT pairs showed an opposite pattern of expression. Therefore, increasing of NAT-related lncRNAs expression can act as positive regulator of stress tolerance in pistachio. Graphical results of gene ontology (GO) enrichment analysis of 76 salt responsive lncRNA-target genes indicated that NAT-related long non-coding transcripts through the regulation of transcription, transcription factors activity, biosynthetic processes, and a response to chitin can act as salt stress responses regulators (Fig. 7).

#### Expression pattern based transcript filtering

**Figure 8:**
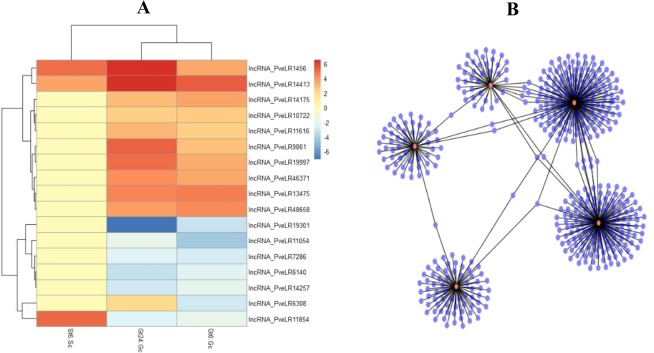
Differentially expressed lncRNAs in pistachio. (**A**) Heatmap showing the expression pattern of 17 selected salt responsive lncRNAs compare to control under salt treatment. Heatmap was generated using the pheatmap package (https://cloud.r-project.org/web/packages/pheatmap/). (**B**) Regulation networks of five selected lncRNAs with the most interaction with their coding target genes. lncRNAs are represented by red nodes, and target genes are represented by blue nodes. The edges represent connections. Interaction networks was generated using the Cytoscape^154^.

**Figure 9:**
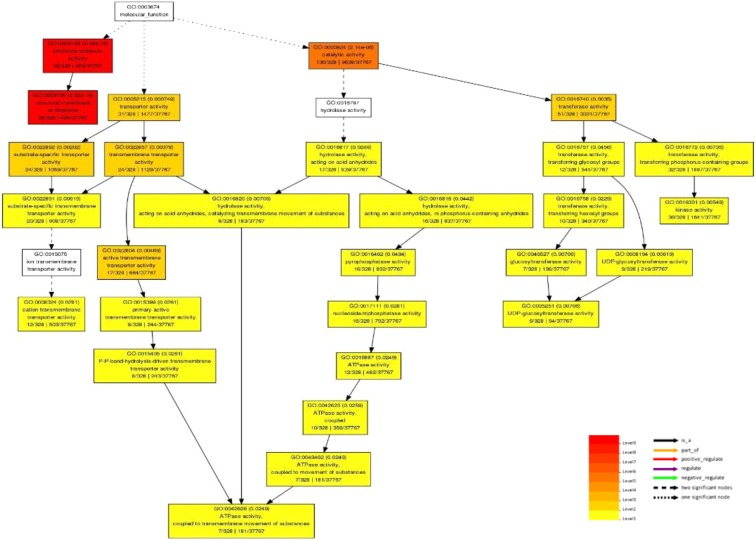
Graphical results of molecular function of potential 446 target genes of five selected lncRNAs that were differentially expressed under salt stress predicted by expression pattern based transcript filtering method. Graphical results were prepared through AgriGO web-based tool^153^.

In some instances, the functional analysis of a particular part of the lncRNAs, not all of them is important to us. In such cases, the use of expression pattern based transcript filtering method is suggested. In this method, lncRNAs filtration was started by drawing of a Venn diagram or other selective analyses. Differentially expressed lncRNAs between Ghazvini as a salt tolerant and Sarakhs as a salt sensitive cultivars are important and can be used to find the reason of successful salt stress tolerance mechanisms in Ghazvini when compared to Sarakhs. To eliminate the differentially expressed lncRNAs that related to genotypic difference in St6-Gt6, Venn diagram was draw between St6-Gt6, Gt6-Gc and Gt24-Gc. Of a total of 199 differentially expressed lncRNAs between Ghazvini and Sarakhs after 6 h salt treatment, 52 and 68 transcripts in St6-Gt6 were commonly differential expressed in Gt6-Gc and Gt24-Gc, respectively. To select St6-Gt6 differential expressed lncRNAs that participate in both early and late responses to salt stress conditions in tolerant cultivar, 17 salt responsive long non-coding transcripts were differential expressed in all three samples are selected for further analysis (Supplementary Fig. S2). Expression pattern of these 17 salt responsive lncRNAs are represented by Heatmap in Fig. 8A. The target genes of these 17 lncRNAs was predicted by Lnc Tar software in salt responsive coding genes of Gt6-Gc and Gt24-Gc. All the 17 studied lncRNAs had target genes but the top five lncRNAs with the most target transcripts were selected for further analysis. The interaction network of these five lncRNAs with their target genes is shown in Fig. 8B. The lncRNA with the biggest number of target genes is lncRNA_PveLR6140, with 155 salt responsive target transcripts. A total of 155, 140, 72, 54 and 45 target genes were identified for lncRNA_PveLR6140, lncRNA_PveLR48658, lncRNA_PveLR1456, lncRNA_PveLR6308 and lncRNA_PveLR14175, respectively. Among these five lncRNAs, lncRNA_PveLR6140 was the only transcript that has been shown down-regulation, while the rest of the lncRNAs are up-regulated in Ghazvini cultivar. Expression analysis of coding target genes of these top five lncRNAs showed that they were upregulated by 60%. Based on gene ontology (GO) enrichment analysis based on molecular function, we found that coding target genes of selected top five salt responsive lncRNAs participate in the cation transmembrane transporter, kinase, UDP-glycosyltransferases and ATPase activity, coupled to transmembrane movement of substances (Fig. 9). Therefore, these gene ontology results related to target genes of top five selected lncRNAs that were differentially expressed between salt tolerant and sensitive pistachio cultivars may suggest a potential mechanism of defense to mitigate the stressed condition in Ghazvini as a salt tolerant cultivar.

In order to study the probability to find miRNA precursor in sequences of top-five selected lncRNAs, we used sequence-structure motif base pre-miRNA prediction webserver (http://www.regulatoryrna.org/webserver/SSMB/pre-miRNA/home.html). Interestingly, the sequence analysis results of these five lncRNAs showed that all of these transcripts had at least one mature miRNA sequences (Supplementary Table S4). Our results suggest that these five lncRNAs can act as a novel miRNA precursor and thus these putative novel miRNAs can play a decisive role in tolerance to salinity in *P. vera*.

**Figure 10:**
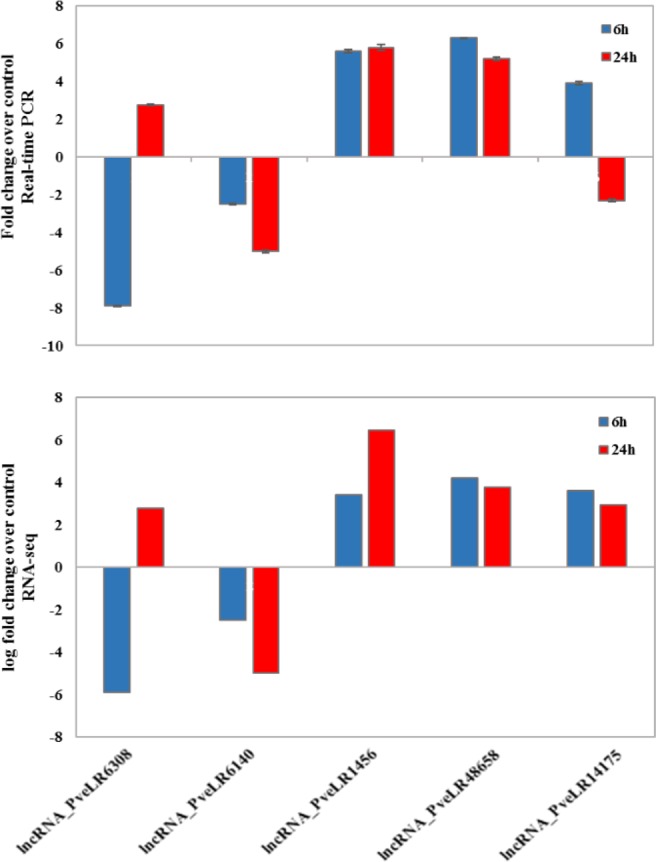
Validation of RNA sequencing results by Real-time PCR. Comparisons between Real-time validation and gene expression profiling data of top five salt responsive selected lncRNAs. The upper and lower graphs represent the Real-time PCR and the RNA-seq results, respectively.

To validate the expression of these top-five lncRNAs under salt stress, qRT-PCR was down to detect their expression changes in roots of Ghazvini cultivar (Fig. 10). Similar to high throughput gene expression profiling data, the qRT-PCR results showed that almost all lncRNAs were successfully validated in control and salt stressed roots. The only exception was the lncRNA_PveLR14175 that its expression at 24 h after NaCl treatment was decreased compared to control. These findings confirm that these lncRNAs are responsive to the salt stress in pistachio roots.

### Identification of miRNA precursor among lncRNA sequences

**Figure 11:**
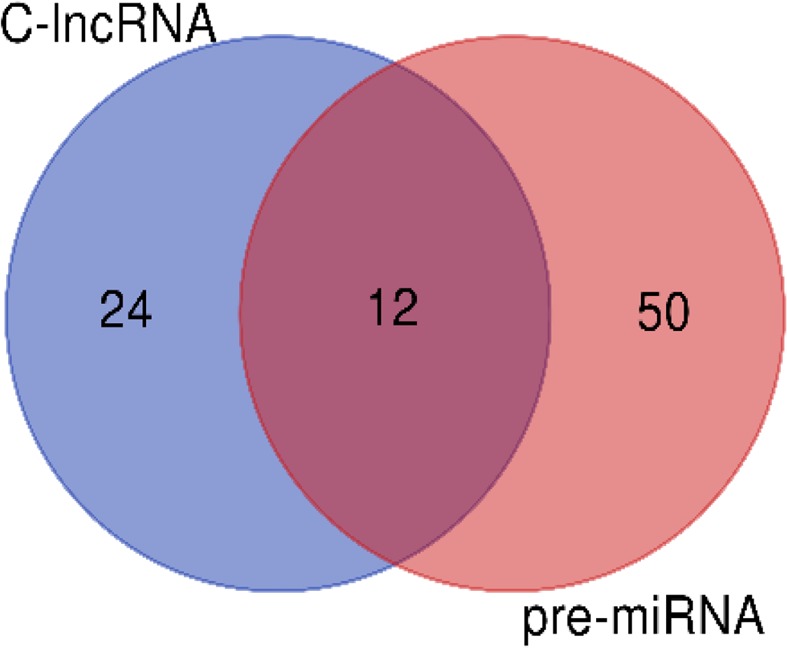
Venn diagrams show the number of common and specific lncRNAs between pre-miRNA and C-lncRNAs.

MicroRNAs (miRNAs) are a class of non-coding-RNAs, with 19–21 nucleotides long, that function as post-transcriptional regulators in eukaryotic genomes. Plant miRNAs are transcribed from intergenic regions by the Polymerase II or III enzymes as primary microRNAs (pri-miRNAs) and then further cleaved to form a precursor-miRNA (pre-miRNA) and finally mature miRNA^73^. Genomic sequence data in pistachio specially about regulatory elements such as miRNAs is very limited. Therefore, identification of miRNA precursor sequences in this plant by use of transcriptomic data can be an important step for further functional studies in this plant. Thus, for this purpose, we aligned miRNA precursor sequences (downloaded from mirBase) against the Pve_lncRNAs; 62 Pve_lncRNAs were similar to known miRNA precursors that they classified as a probable pre-miRNA in *P. vera*. These results indicate that pistachio miRNA precursors have somewhat evolutionary conservation among other species. In this study, some of the conserved miRNA precursor was identified in *P. vera*, but other conserve and novel miRNA precursors are still remaining to identify. As illustrated in the Venn diagram (Fig. 11), pistachio have 12 common transcripts between pre-miRNAs and C-lncRNAs (Conserved lncRNAs). The number of common transcripts between pre-miRNAs and C-lncRNAs indicated that 33% of Pve_lncRNA conservation was related to conservation of pre-miRNAs.

### Salt-responsive miRNA-related functions of lncRNAs

**Figure 12:**
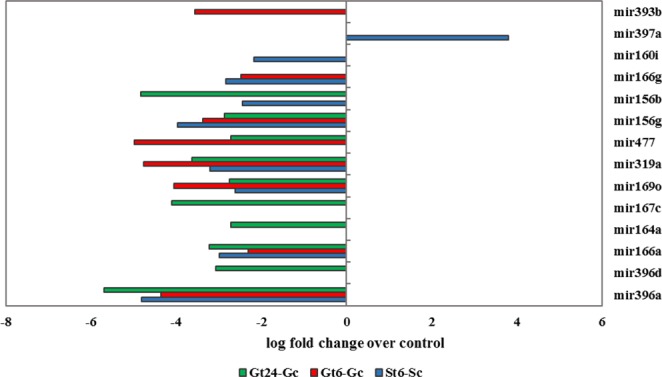
The log fold change value of 14 miRNA-lncRNAs that differential expressed under salt stress in St6-Sc, Gt6-Gc and Gt24-Gc.

Under environmental stress, the expression change of miRNA can regulate gene expression at the post-transcriptional level by interacting with the complementary binding sites on target genes. miRNAs are either up-regulated by stress, where they enhance the suppression of the genes serving as negative regulators of stress tolerance or down-regulated where the target gene is accumulated and acts as positive regulators of stress^74^. To explore such functional roles of miRNAs, miRNA precursor prediction in differentially expressed sequences of St6-Sc, Gt6-Gc and Gt24-Gc was performed. miRNA identification process leads to the identification of 9, 8 and 10 pre-miRNAs belonging to 7, 7 and 8 miRNA families in St6-Sc, Gt6-Gc and Gt24-Gc, respectively. The names of these pre-miRNAs plus the corresponding lncRNA-IDs are given in Table 1. The log fold change value of these differential expressed miRNA-lncRNAs was shown in Fig. 12. As you can see in Fig. 12, all pre-miRNAs, except mir397a, show down-regulation under salt stress condition.

**Table 1:** The names and IDs of differential expressed miRNA-lncRNAs in different samples under salt treatment.

Pre-miRNA differential expression					
Gt6-Gc		Gt24-Gc		St6-Sc	
lncRNA ID	miRNA name	lncRNA ID	miRNA name	lncRNA ID	miRNA name
lncRNA_PveLR15531	mir396a	lncRNA_PveLR15531	mir396a	lncRNA_PveLR12931	mir166g
lncRNA_PveLR12931	mir166g	lncRNA_PveLR14254	mir396d	lncRNA_PveLR14505	mir166a
lncRNA_PveLR14505	mir166a	lncRNA_PveLR14505	mir166a	lncRNA_PveLR3337	mir160i
lncRNA_PveLR6504	mir393b	lncRNA_PveLR10955	mir164a	lncRNA_PveLR6753	mir169o
lncRNA_PveLR6753	mir169o	lncRNA_PveLR8715	mir167c	lncRNA_PveLR13016	mir319a
lncRNA_PveLR5421	mir477	lncRNA_PveLR6753	mir169o	lncRNA_PveLR17262	mir156g
lncRNA_PveLR13015	mir319a	lncRNA_PveLR13015	mir319a	lncRNA_PveLR3941	mir156b
lncRNA_PveLR17263	mir156g	lncRNA_PveLR5421	mir477	lncRNA_PveLR8830	mir397a
		lncRNA_PveLR17263	mir156g	lncRNA_PveLR15531	mir396a
		lncRNA_PveLR3941	mir156b

**Figure 13:**
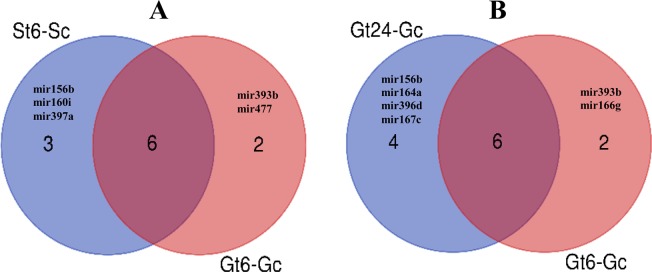
Venn diagram showing common and specific lncRNAs between different samples. (**A**) Venn diagrams show the common and specific differentially expressed miRNA-lncRNAs between St6-Sc and Gt6-Gc and also (**B**) between Gt6-Gc and Gt24-Gc.

**Figure 14:**
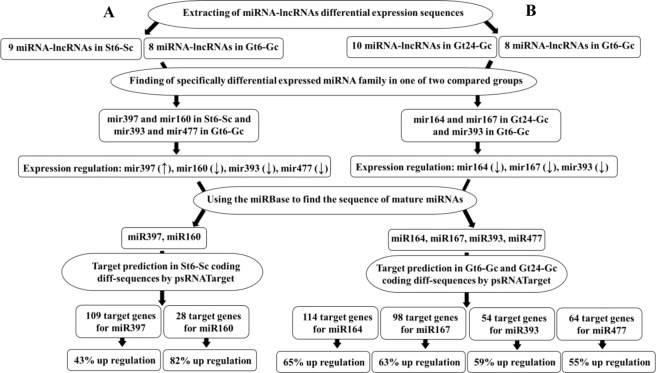
The pipeline for the identification and target prediction of differential expressed miRNA-lncRNAs. (**A**) Identification and salt responsive target prediction of specifically differential expressed miRNA family between St6-Sc and Gt6-Gc. (**B**) Identification and salt responsive target prediction of specifically differential expressed miRNA family between Gt24-Gc and Gt6-Gc.

**Figure 15:**
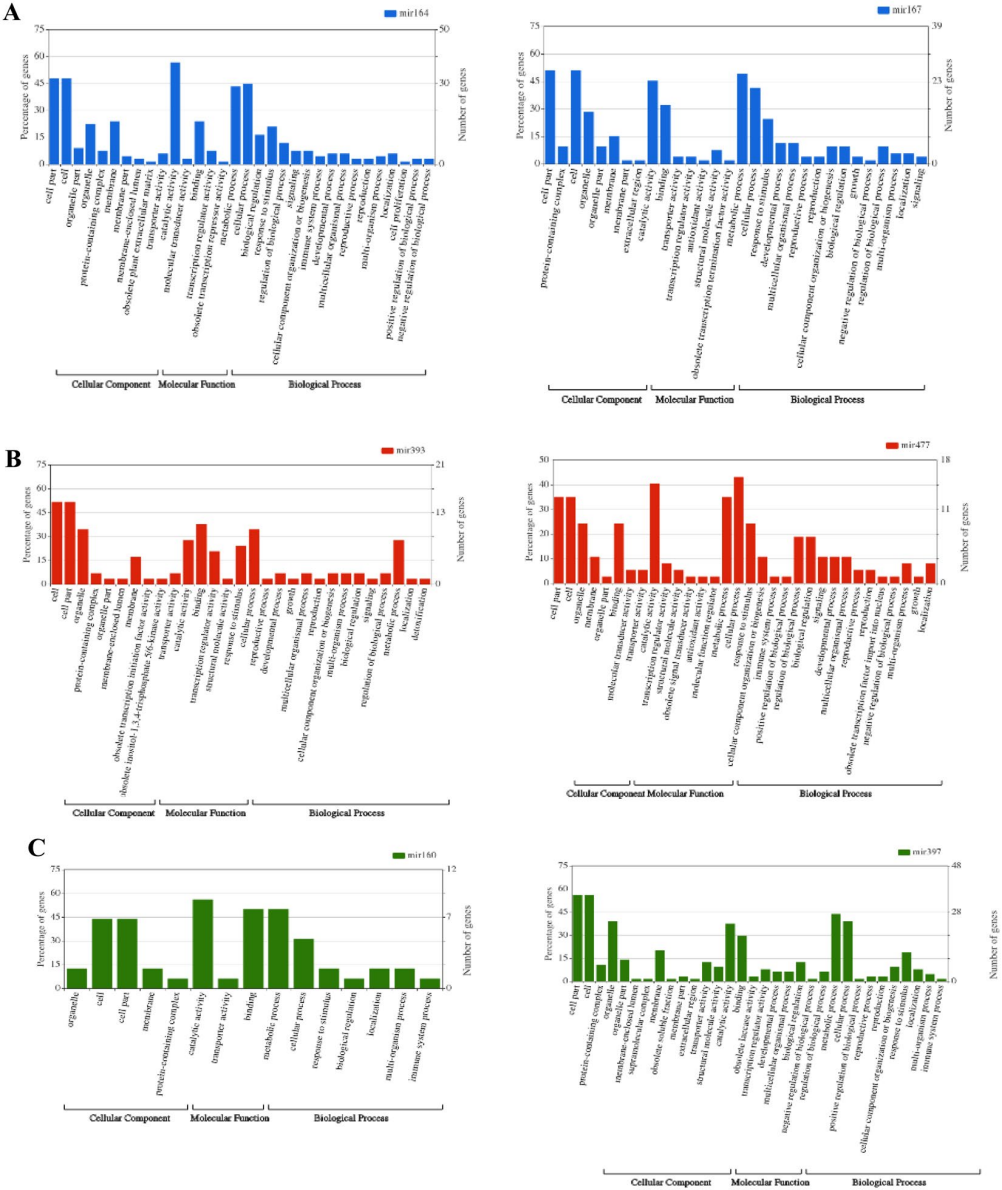
Gene ontology distribution results of coding target genes of selected miRNA-lncRNA family. (**A**) Targets of mir164 and 167 (**B**) Targets of mir393 and 477 (**C**) Targets of mir160 and 397. GO terms histogram was prepared through WEGO online tool^159^.

To investigate the relationship between miRNA-lncRNA expression changes and sensitive and tolerant cultivars function under salt stress condition, miRNA with specific expression in St6-Sc, Gt6-Gc and Gt24-Gc was identified (Fig. 13A,B). We used the mirBase to find mature miRNA sequences of specifically differentially expressed miRNA-lncRNA families. In the next step, target prediction of mir397 and mir160 as specifically differential expressed miRNA families in St6-Sc was down using target file of St6-Sc_coding diff-sequences by psRNATarget. A total of 109 and 28 salt responsive coding target genes was found for mir397 and mir160, respectively. The target analysis of mir393 and mir477 families (specific diff-expressed in Gt6-Gc) and also mir164 and mir167 families (specific diff-expressed in Gt24-Gc) was down with searching of target genes by psRNATarget in Gt6-Gc_coding and Gt24-Gc_coding differential sequences, respectively. A total of 54, 64, 114 and 98 salt responsive coding target genes was found for mir393, mir477, mir164 and mir167, respectively. (Fig. 14). The putative functions of coding target genes for each of these 6 miRNA-lncRNAs were analyzed by agriGO (Fig. 15). In molecular function section, having binding site can lead to transcription regulation and finally generate protein with specific catalytic activity to set up stress response mechanisms. These molecular functions form the specific metabolic and cellular processes that can ultimately lead to response to stimulus. These molecular functions and biological process were the top three enriched gene ontology term in Ghazvini as a tolerant cultivar but in Sarakhs, transporter activity replace the transcription regulator activity under salt treatment. Moreover, the other difference between miRNA-lncRNA target genes of Ghazvini and Sarakhs is that more than 50% of coding target genes of mir397 were down-regulated by salt stress in Sarakhs but in Ghazvini, all target genes of 4 specific differential expressed miRNA families had more than 50% up-regulation (Fig. 14). These results suggest that the inductive role of miRNAs in regulation of these functions and process might be involved in salt stress tolerance of Ghazvini.

Identification of putative miRNA target genes may be a predictive analysis and is not completely accurate. Therefore, in such a situation, given that the high-throughput sequencing technology generate the high volume of data, use of percentage can be very important and decisive. Interestingly, in the present study, mir164, mir167, mir393, mir477 and mir160 with down-regulation under stress condition, act as a positive regulator of salt stress tolerance that induce up-regulation of their coding target genes by more than 50%. On the other hand, the only miRNA (mir397) with up-regulation under stress condition, act as a negative regulator of salt stress tolerance that leads to less than 50% up-regulation in their target genes (Fig. 14).

**Figure 16:**
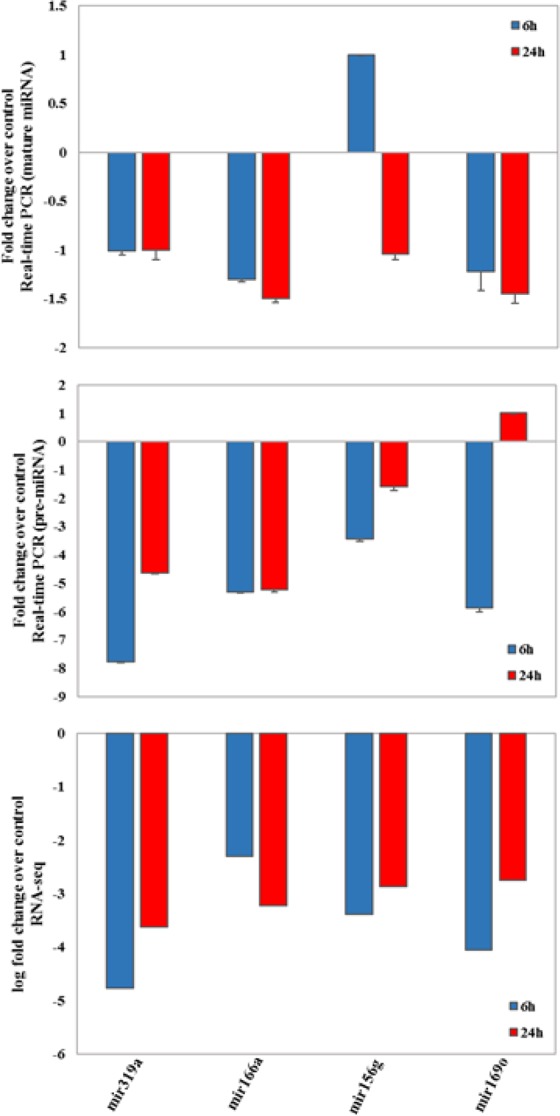
Validation of RNA sequencing results of miRNA-lncRNAs by Real-time PCR. Comparisons between Real-time validation and gene expression profiling data of four salt responsive selected miRNA-lncRNAs. The two upper and one lower graphs represent the Real-time PCR and the RNA-seq results, respectively.

To validate the expression of putative pre-miRNAs, the expression of four differential expressed miRNA-lncRNAs and their mature miRNAs were detected by qRT-PCR in roots of Ghazvini cultivar. Similar to high throughput gene expression profiling data, the qRT-PCR results showed that almost all miRNA-lncRNAs and mature miRNAs were successfully validated in control and salt stressed roots. The only exceptions were the up-regulation of mir156g (6 h) and mir169o (24 h) in mature and pre-miRNAs, respectively. Our findings indicate that these miRNA-lncRNAs are likely to be precursors of miRNAs and they are responsive to salt stress in *P. vera* (Fig. 16).

## Discussion

The majority of well-studied lncRNAs are belong to human and animals, while the biological functions of most plant lncRNAs are largely unknown. The results of these studies show that plant lncRNAs are involved in stress responses. In plants, lncRNAs may execute their function to respond to stresses in five different ways including target mimicry, sRNA precursors, interaction between sense mRNA and antisense lncRNA (NAT pairs), lncRNA-mediated chromatin modification and RNA-directed DNA methylation^37^.

The increased effects of salt stress, caused by climate change, compel the need to improvement of economic crop species such as pistachio as a one of the most important nut crop. To achieve this goal, genomic and transcriptomic information are required. However, there is paucity of such information on pistachio. Therefore, transcriptomic studies can help to gain insight into the sequences and functions of the non-coding and coding-RNAs in this plant. Here, we conducted a transcriptome-wide identification of lncRNAs and constructed a reference lncRNA sequences for further analysis. Calculating GC content of the reference lncRNAs revealed an average of 36%. GC content varies strongly among species both on average, from about 13 to 75% in Eukaryotes, and in heterogeneity, some genomes being homogeneous whereas others are highly heterogeneous^75,76^. The GC content comparison between non-coding and coding sections of pistachio transcriptome showed the GC content heterogeneity. The average GC content of lncRNAs was 4% lower than coding-RNAs. Such a result was also reported in other plants including wheat (with a difference of 8%)^60^ and Miscanthus (with a difference of 5%)^66^.

Differential expression analysis leads to identification of stress responsive lncRNAs in two salt tolerant and sensitive cultivars of *P. vera*. The dynamic changes of lncRNAs expression during stress conditions indicated that lncRNAs might play significant regulatory roles at transcriptional and post-transcriptional levels in response to salt stress. In the present study, we provide model for functional annotation of lncRNAs in non-model plant species using two methods. First method was based on initial filtration of non-coding transcripts by blasting. Due to the difference between the length of the non-coding and coding transcripts, the simple filtering criteria based on continuous matching region longer than 150 nucleotides with ≥90% identity was used for identification of NAT pairs between non-coding and coding section of the transcriptome. We identified 143 salt responsive lnc-coding RNA-NAT pairs in Ghazvini as a salt tolerant pistachio cultivar. lncRNA-mRNA hybridization analysis confirmed the interaction of 76 lncRNA-mRNA pairs. As can be observed, the results of the Lnc Tar software are more than 50% (53%) consistent with the results of the NAT prediction (Supplementary Fig. S2). Therefore, BLAST-based initial filtration method can be useful to functional analyze a great number of lncRNAs and eliminate non-NAT related lncRNAs in the early stages of analysis. High similarity (89.5%) of expression pattern between lncRNA-mRNA pairs indicates that there is a direct relationship between the expression of lncRNAs and their target genes in lnc-coding RNA-NAT pairs. Interestingly, the results of target gene enrichment in GO categories associated to gene regulation (i.e., ‘regulation of transcription’ and ‘transcription factor activity’) and to stress response (i.e., ‘response to stimulus’ and ‘response to chitin’) (Fig. 7). Moreover, among the coding-target genes (Supplementary Table S5) in the pathway of gene expression regulation, we found a set of transcription factors families such as GRAS (targeted by lncRNA_PveLR31442), WRKY (targeted by lncRNA_PveLR32945) and bZIP (targeted by lncRNA_PveLR5534). Genome-wide analysis of lncRNAs in bananas under cold stress revealed that many of lncRNA targets were transcription factor genes, such as MYB, WRKY, bZIP and GRAS transcription factors^77^. Similarly, the co-expression analysis of lncRNAs with transcription factors indicated that the transcription factors related to the WRKY, NAC, MYB and bZIP families were enriched in salt stress condition in wheat^78^. Several reports indicated that all these transcription factors are involved in response to salinity stress, for example numerous studies have established the important roles of WRKY proteins in various physiological processes such as seed germination^79^, lateral root formation^80^, flowering time^81^, fruit ripening^82^, leaf senescence^83^, and metabolic processes^84,85^. In addition, WRKY protein act as an important regulator in plant stress responses against various abiotic stresses, such as salinity^86^. The regulation of transcription factors by lncRNAs has been recently reported in cotton. It was shown that lncRNA973 may regulate the expression of transcription factor WRKY46. The expression of AtWRKY46 increased in OX-lncRNA973 and decreased by lncRNA973 silencing and significant up-regulation of OX-lncRNA973 was observed in plants under salt treatment^87^. The activation of lncRNAs by transcription factors has also been indicated in tomatos. It was reported that activation of lncRNA33732 expression by WRKY1 through sequence-specific interactions in the lncRNA33732 promoter during *Phytophthora infestans* infection can induce early defense responses^88^. Also, the basic region-leucine zipper (bZIP) family as one of the most conserved transcription factors, have been found to mediate various biological processes, such as cell elongation^89^, organ and tissue differentiation^90,91^ and energy metabolism^92^. Moreover, the bZIP transcription factors play a role in plant responses to biotic and abiotic stresses such as salt and drought stress conditions^93,94^.

In the pathway of response to stimulus among the predicted target gene of NAT pairs, Chitin elicitor receptor kinase 1 (CERK1) gene (targeted by lncRNA_PveLR32819) is present to set up signaling pathways to response to salt stress. In Arabidopsis, the physical interaction of CERK1 with ANNEXIN 1 (ANN1) procedure a calcium-permeable channel in NaCl-induced [Ca2+]cyt signaling pathway under salt stress condition. Thus, the cerk1 mutant was more salt sensitive than the wild type under NaCl treatment^95^. In accordance with our results, differentially expressed genes were also found to be enriched in ‘chitin binding’ in pistachio under salt stress condition^36^.

In addition, among the predicted target gene of NAT pairs, we found protective genes against stress damages such as Late embryogenesis abundant (LEA) (targeted by lncRNA_PveLR53178) and Laccase (targeted by lncRNA_PveLR34269) genes. LEA proteins are involved in protection against desiccation during seed dehydration and acts as a dewatering protectant in vegetative tissues under stress conditions^96^. They play a role in protecting molecular and cellular structures via the renaturation of unfolded proteins and ion sequestration that lead to the protection of proteins and membranes from the damaging effects of water stress^97^. Laccases, as multi-copper-containing glycoproteins, catalyze the polymerization of the monolignol alcohols in the lignin biosynthesis pathway. Different stresses in plants can cause the accumulation of reactive oxygen species (ROSs), accompanied by the accumulation of lignin. Therefore, lignin metabolism is important in plant responses during biotic and abiotic stresses such as drought and salt stress conditions^98^. Important developmental processes and responses to biotic or abiotic stresses are regulated by different hormones in plants^99^. In addition, cross-talks among different hormonal pathways under stress conditions are very important in abiotic stress tolerance^99,100^. Genes involved in the signaling pathway of hormones such as abscisic acid (ABA), jasmonic acid (JA), and ethylene are among the coding-target genes of lnc-coding RNA-NAT pairs. ABA is involved in the signaling of water stress, such as salinity and drought conditions that cause the activation of water-saving mechanisms such as stomatal closure^101,102^ and the expansion limitation of leaves^103^. It was reported that ethylene receptors are involved in ABA production and lead to the increased survival of rice plants under salt stress condition^104^. Jasmonate ZIM-domain (JAZ) family proteins targeted by Pve_lncRNAs in NAT pairs are involved in regulating different biological processes in plants. The transcriptome analysis of cotton showed that GhJAZ2 may regulate stress responses by participating in α-linolenic acid metabolism and jasmonate signaling^105^. Through comparative transcriptome analysis, a similar result was obtained in pistachio. This result indicated that the biosynthetic pathway of jasmonic acid (JA) plays an essential role in salt stress tolerance in pistachio^36^. Therefore, it can be concluded that lncRNAs in NAT pairs use different ways to modulate salt stress tolerance responses in pistachio seedlings including gene expression regulation of transcription factors, LEA, Laccases, and CERK1, and controlling the signaling pathways of plant hormones.

Second method was based on the initial selection of a specific section of lncRNAs for functional annotation. In this method, lncRNAs filtration was started by drawing of Venn diagram or other selective analyses. One of the goals of this study was that to find the cause of the differences between the salt responses of two salt tolerant and sensitive pistachio cultivars. For this purpose, differentially expressed transcripts can be helpful. A total of 17 non-coding transcripts that differential expressed between Ghazvini and Sarakhs and also participating in both early and late responses to salt stress conditions in Ghazvini were selected. After target gene prediction by Lnc Tar software, top five lncRNAs with the most target transcripts are selected for further analysis. Gene ontology (GO) enrichment analysis of these top five salt responsive lncRNAs (Fig. 9) indicated that their coding target genes are participate in the cation transmembrane transporter, kinase, UDP-glycosyltransferase and ATPase activity, coupled to transmembrane movement of substances. The plasma membrane H^+^-ATPase in plants acts as the primary transporter that hydrolyze ATP to proton transport. This transport creates proton motive force that is used by secondary transporters. In salt tolerance mechanisms, in order to avoid salt accumulation in the cytosol, secondary transporters participate in ion homeostasis and metabolites transport. A response to the accumulation of toxic ions in the cytosol is their compartmentalization within the vacuole or transferring them out of the cell. For this action, a Na^+^/H^+^ antiport is involved and thus activation of this transport by a vacuolar and plasma membrane H+-ATPase is necessary^106^. Family-1 glycosyltransferases are also named UDP-glycosyltransferase (UGTs). They are the biggest glycosyltransferases family in plants that transfer uridine 5′-diphosphate sugars onto different molecules and modulate different metabolic processes. It was reported that UGT79B2 and UGT79B3 act as anthocyanin rhamnosyltransferas and confer abiotic stress such as salt stress tolerance through anthocyanin accumulation in Arabidopsis^107^. Anthocyanins, an important class of plant flavonoids, play roles in vegetative tissues to absorb UV and high light irradiation and scavenging of ROSs^108^. Regarding the role of H^+^-ATPase and cation transporter in ion homeostasis, kinase in stress-related signaling^109^ and anthocyanin in ROS scavenging under salinity stress, the difference in the expression of these top five selected lncRNAs that are involved in regulating the activity of these genes and pathways may be the key factor in increasing the tolerance of Ghazvini to surpass the salt stress condition when compared to Sarakhs. On the other hand, these results indicate that the regulation of these genes both in early and late responses to stress condition is very important for salt stress tolerance. Surprisingly, the sequence analysis of these five lncRNAs for predicting of miRNA precursor reveals that all of these transcripts have at least one mature miRNA sequences. The role of these five putative novel miRNA precursors should be further characterized so as to identify the regulatory mechanism of their target genes associated with stress tolerance in pistachio. The top five salt responsive lncRNAs were selected to confirm experimentally by qRT-PCR. These five lncRNAs showed a significant change in their activity in roots under salt stress, in accordance with our RNA-seq analysis.

**Figure 17:**
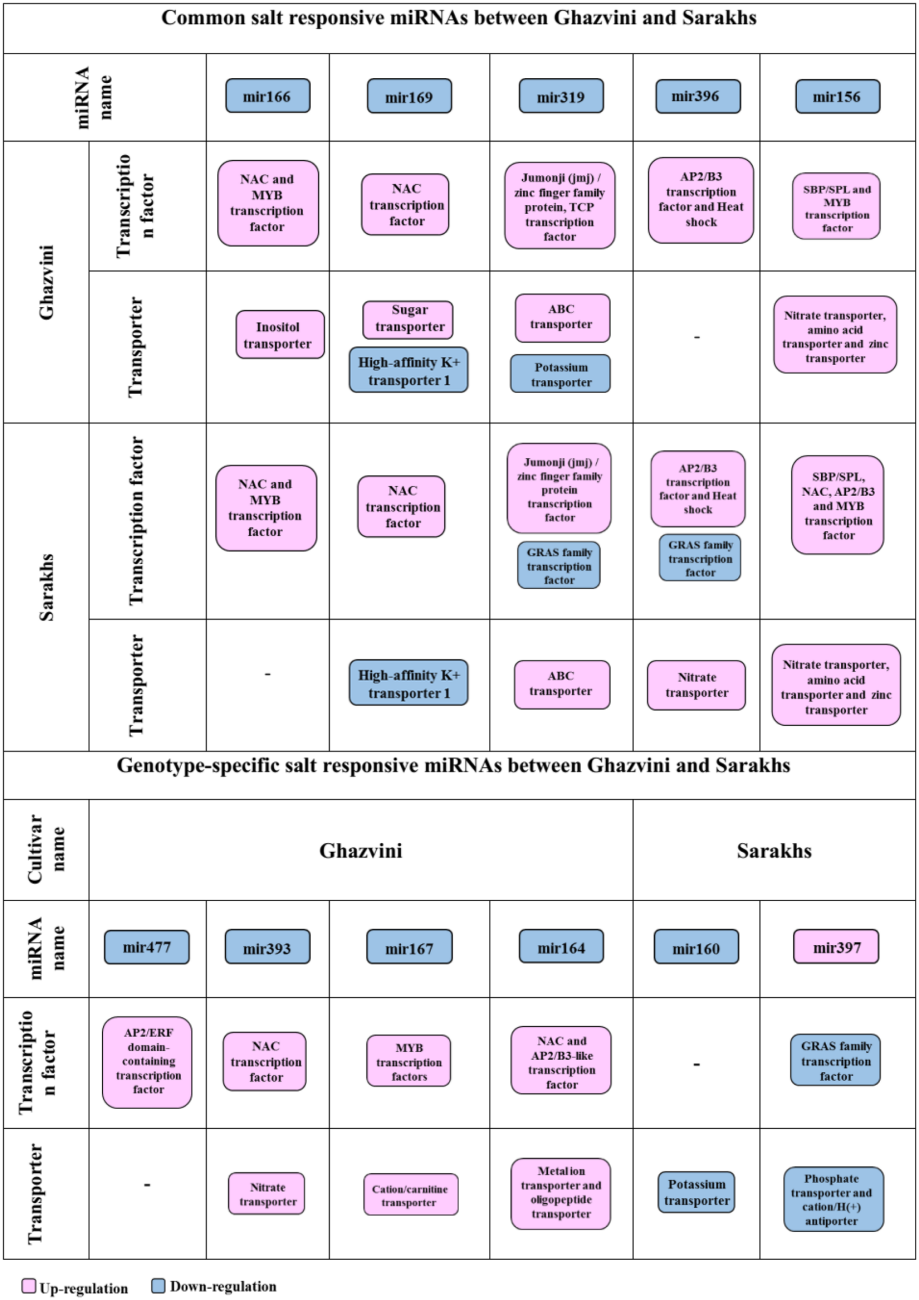
Common and genotype-specific salt responsive miRNAs between Ghazvini and Sarakhs and their target genes (transcription factor and transporter).

We also the probability of founding miRNA precursors in lncRNA sequences was investigated. miRNAs are important classes of ncRNAs that play a role in gene expression regulation in plants. In Populus, among 410 gibberellin-responsive lncRNAs, 10 miRNA precursors were identified^110^. In maize, 8 out of 664 drought-responsive lncRNAs were identified as known maize miRNA precursors^49^. From the complete dataset of Pve_lncRNAs, we identified 62 potential miRNA precursors, which are conserved among other plant species. Certainly, there are many unknown miRNA precursors among pistachio lncRNAs that related to novel miRNAs and should be identified in the future. It has been reported that some miRNAs are stress responsive and could be involved in the plant responses to abiotic stresses such as salt stress^111^. In the present work, 14 salt-responsive miRNA precursors belonging to 11 families were detected in two pistachio cultivars. The target genes of stress-responsive miRNAs can be a transcription factor or functional gene. The target genes of these 11 salt-responsive miRNA precursor families with a transcription factor or transporter function are shown in Fig. 17. Non-genotype-specific salt responsive pistachio miRNAs are belonging to mir160, mir169, mir319, mir396 and mir156 families that were similarly regulated between the two pistachio cultivars under salt stress; they were down-regulated, indicating that probably they are positive regulators of salt tolerance, resulting in the accumulation of their target transcripts in response to stress condition. The search for target genes in salt responsive coding section of transcriptome shown that these miRNAs similar to genotype-specific salt-responsive miRNAs, up-regulated their coding target genes by more than 50% in the two studied cultivars. The genotype-specific salt-responsive miRNAs might explain the difference between stress responses of two salt tolerant and sensitive pistachio cultivars. The early genotype-specific salt responsive miRNAs are mir169 and mir397 in Sarakhs and mir393 and mir477 in Ghazvini. The late-specific salt responsive miRNAs in Ghazvini are mir164 and mir167. The diversity of miRNAs is high to specifically match nearly any given RNA encoded in a genome, consequently regulating almost every aspect of plant biology, often act as upstream regulators of genes through influencing control by transcription factors^111^. As can be observed in Fig. 17, there are various transcription factors in the target genes of pistachio miRNAs. Among these transcription factors AP2/ERF, MYB and NAC have been demonstrated to play an important regulatory role in plant responses to abiotic stresses^112–114^. NAC-domain proteins as a transcription factor were reported to be salt induced in many species^115,116^. NAC-domain proteins are unique to plants, and their transcripts are predicted as a target of mir166, mir169, mir393 and mir164 in pistachio. In Arabidopsis, NAC1- overexpressing lines were bigger plants when compare to the wild type with thicker stems, larger leaves and more roots. It was thought that NAC1 might be act as an early auxin-responsive transcription factor to promote lateral root development^117^. At the early stage of salt stress, mir166 and mir169 targeted the NAC-domain proteins in two studied pistachio cultivars but in addition to the common miRNAs, in salt-tolerant pistachio cultivar, genotypic-specific salt responsive mir393 at the early and mir164 at the late stage of salt stress targeted NAC-domain proteins. In accordance with our findings, mir164 targeted NAC transcription factor during drought stress in cassava and lateral root development in tobacco^118,119^ and also mir166 targeted NAC transcription factor during fibrous and storage root development in sweet potato^120^. It can be concluded that Ghazvini might change their morphological characteristics in a different way than the sensitive cultivar to enhance root and shoot development to counteract with the damage caused by salt shock. The MYB transcription factor contains a MYB domain that is highly conserved across all eukaryotes^116^. Numerous studies have suggested that MYB proteins are also involved in regulating plant responses to biotic and abiotic stresses^121–123^. Overexpression of TaODORANT1 enhances resistance to drought and salt stress in transgenic plants^123^. Moreover, PbrMYB21 gene by regulating of polyamine synthesis pathway plays a role in drought tolerance mechanisms of plants^124^. In the present study, MYB transcripts are predicted to be a target of mir166, mir156 and mir167 in pistachio. In accordance with our results, mir156 in switchgrass and mir166 in peanut targeted MYB transcription factor during drought stress^125^ and disease resistance^126^, respectively. AP2/ERF transcription factor family has introduced as important regulators of several abiotic stresses and respond to multiple hormones. Gene expression profiling studies under normal and stress conditions have indicated that the expression of most AP2/ERFs genes are low under normal conditions, whereas their expression can be regulated by stress conditions that leads to the control of multiple plant stress responses, including plant growth^127^. Among salt responsive miRNAs in pistachio, mir477 targeted AP2/ERF transcription factor.

The difference between the target genes with a transcription factor function of salt responsive miRNAs are not high and is limited to the up-regulation of TCP (target gene of mir319) in Ghazvini and down-regulation of GRAS (target gene of mir319 and 396) in Sarakhs. TCP transcription factor are involved in so many important developmental processes and their up or down-regulation are reported in response to stress conditions^128^. In rice, the transcript of the class I TCP OsTCP19 is up-regulated during salt and water-deficit stresses. In Arabidopsis, overexpression of OsTCP19 increased abiotic stress tolerance and such as plants are reported to grow better under osmotic stress^129^. It was reported that TCP transcription factor was targeted by mir319 under salt stress condition in *Arabidopsis thaliana*^130^ and *Solanum linnaeanum*^131^. GRAS family transcription factors as a specifically differentially expressed miRNA target gene in Sarakhs, play important roles in different physiological processes, such as root radial shape, axillary meristems evolution, gibberellin signal transduction, signaling of phytochrome and detoxification^132–134^. A large number of genes in GRAS family are involved in responses to stress conditions^135^. For example, PeSCL7 gene as a member of GRAS family in Poplar plant, was reported to increase salt and drought tolerance in transgenic Arabidopsis plants^136^. These two transcription factors play a role particularly in morphological adaptation during salt stress condition but this adaptation has probably been more successful in Ghazvini (Fig. 17).

In the section of common salt responsive miRNAs between two cultivars, down-regulation of HKT1, targeted by mir169 was visible. The high-affinity K^+^ transporters (HKTs) have specificity for Na^+^ (class I), and Na^+^ and K^+^ (Class II). Thus, regarding the role of this transporter as a one way to uptake of Na^+^, expression regulation of this gene is important under salt stress condition. HKT1-type transporters play a role in maintaining balance between sodium and potassium ions under salt stress to prevent sodium ion toxicity in the cell. In rice, salt tolerance of plant was increased by down-regulating of HKT1 gene^136,137^. In addition to HKT1, ABC transporter in two pistachio cultivars was up-regulated and targeted by mir319. ABC is a stress-related secondary metabolites transporter such as alkaloids, terpenoids, polyphenols and quinines and hormones such as auxin and abscisic acid^138^. In Arabidopsis, ABC transporter affected Na^+^/K^+^ homeostasis and play a role in response to salt stress^138^. Moreover, regulation of ABC transporter by miRNA for ion homeostasis was reported in *Oryza glaberrima* under salt treatment^139^. Another up-regulated transporter in two studied cultivars is nitrate transporters. Nitrate transporters have been reported to act as a bridge between different hormones such as indole-3-acetic acid, ethylene and abscisic acid in response to stress conditions. For example, in Arabidopsis NPF2.3, a NO3- efflux transporter, is involved in NO3- translocation from roots to shoots in response to salt stress^140^. In non-genotype-specific differentially expressed miRNAs, the significant difference between target genes in the section of transporters of two studied cultivars was related to inositol and sugar transporters in Ghazvini. In some plant species, inositol is used for the production of the compatible solutes pinitol and ononitol during stress conditions such as salt stress^140,141^. In addition, Sugar transporters play an important role in abiotic stress tolerance because they transport carbohydrate within the cells which can act as stress-related signal or compatible solute and also transport the sugar from source to sink that the regulation of this processes is crucial for plant tolerance to stressful condition^142^. From these findings, it can be concluded that Ghazvini have used different mechanisms compared to Sarakhs for carbohydrate partitioning and compatible solutes selection and compartmentation under salt treatment, which ultimately leads to greater success in coping with salt stress. In genotype-specific differentially expressed miRNAs, the significant difference between target genes in the section of transporters was related to cation/carnitine transporter targeted by mir167 in Ghazvini. Carnitine as a ubiquitous quaternary ammonium compound that is found in all living organisms, is participated in some biological functions such as energy metabolism, and response to stress conditions. Carnitine treatment increased the germination and survival rates of plants grown under salt stress. A transcriptome gene expression analysis of seedlings treated with exogenous carnitine indicated that carnitine regulated the expression of genes related to drought stress^143^. This result indicated that the use of genotype-specific miRNAs and consequently induction of specific target genes such as cation/carnitine transporter to adjust the responses to salinity stress that might result in different salt sensitivities among two pistachio cultivars (Fig. 17). The four salt responsive putative pre-miRNAs and their mature miRNAs were selected to confirm experimentally by qRT-PCR. The expression of these miRNA-lncRNAs and mature miRNAs were changed significantly in roots of Ghazvini cultivar by salt stress, in accordance with our RNA-seq analysis but as can be observed in Fig. 16, mature miRNAs generally show lower expression levels than miRNA-lncRNAs.

The results of present study indicated that lncRNAs have an important role in both transcriptional and post-transcriptional regulation level and also can regulate transcription factors such as MYB, NAC, WRKY, bZIP, TCP and GRAS and functional genes such as transporters, ATPase, LEA, Laccase, CERK1, UGT and genes involved in the hormone signaling pathways in the pistachio adaptation to high concentration of salt. Given that the biological function of the most lncRNAs is unknown, BLAST-based filtering, expression based filtering, identification of conserve and novel miRNA precursor, prediction and analysis of salt responsive coding target genes approaches were used in this study in order to increase our knowledge about the functions of lncRNAs. Our findings constitute a comprehensive resource of pistachio lncRNAs and also provide a valuable stress responsive lncRNAs and miRNAs for future research in this direction. Due to the important regulatory role of lncRNAs, it is necessary to use lncRNA sequences to create a new generation of lncRNA related markers for improvement of valuable crop.

## Methods and Materials

### Data collection

In order to screen the NaCl-sensitive and NaCl-tolerant pistachio cultivars, we selected five pistachio cultivars, including *P. vera* L. cv. Sarakhs, Badami-zarand, Ghazvini, Akbari and Kaleghuchi. The 6-week-old hydroponically grown plants were exposed to 250 mM NaCl. For selection of tolerant and sensitive cultivars, Malondialdehyde, plant survival rate, sodium and potassium content was measured after 8 days’ salt treatment. Finally, salt sensitive (Sarakhs) and tolerant (Ghazvini) pistachio cultivars were determined^35^.

For transcriptome sequencing, 6-week-old hydroponically grown *P. vera* L. cv. Sarakhs (as a salt sensitive) and Ghazvini (as a salt tolerant), were treated by 250 mM NaCl. At four time points, including 0, 6, 24, and 48 h after salt treatment, the stems, roots, and leaves of pistachio plants were collected separately. The total RNA was extracted separately from 24 samples including two pistachio cultivars, four time points after salt treatment and three types of tissue (stems, roots and leaves). RNA samples were then pooled for comprehensive transcriptome sequencing^35^. Furthermore, the RNA sequencing of six RNA samples including three time points (0, 6 and 24 hours after salt stress (250 mM NaCl)) of 6-week-old salt-tolerant and salt-sensitive pistachio cultivars roots was done using the Illumina Hiseq 2000 platform. Differentially expressed genes between salt-tolerant and salt-sensitive pistachio cultivars and compared to the control were identified using edegR package (Unpublished data).

### Plant materials and salinity treatment

The pistachio seeds (Sarakhs and Ghazvini cultivars), were obtained from the Pistachio Research Center. Seeds were surface-sterilized with 20% commercial bleach for 20 min and Captan 0.3% (fungicide) for 60 min, rinsed thoroughly with distilled water. After chilled at 4 °C for 14 days, seeds were grown in boxes containing Hoagland’s nutrient solution (pH = 5.8) with 16-h light/8-h dark photoperiods at 25 °C. After 6 weeks, plants were incrementally treated with NaCl up to 250 mM to the hydroponic culture medium. The roots of pistachio plants were rinsed with distilled water and then collected at 0, 6 and 24 h after NaCl treatment and immediately frozen in liquid nitrogen. We used three biological replicates.

### Transcriptome assembly

FastQC tool (http://www.bioinformatics.babraham.ac.uk/projects/fastqc/) was used for quality control of raw reads, the reads were pre-filtered to remove probable contaminants. Adapter sequences and low quality tags in the raw data were trimmed by Trimmomatic software (v0.38)^144^. Ribosomal RNA data was also removed from the remaining data by alignment. The clean reads to construct a reference transcriptome were assembled de novo using Trinity software (v2.8.4)^145^ with default settings. All transcripts longer than 200 bp were retained. Trinity isoforms were clustered using CD-HIT-EST^146^ with 90% identity to eliminate redundancies.

### Identification of lncRNAs

To filter the coding transcripts, we firstly aligned (BLASTx) all transcripts over 200 bp against the UniProt/UniRef90^147^ and the NCBI/NR protein database using locally installed NCBI-BLAST software (v2.8.1+)^148^ with the e-value threshold of 1e-5. Sequences matching with proteins were excluded from further analysis. The coding potential of remaining sequences had evaluated using Transdecoder (v5.5.0)^145^ and CPC (version 1)^149^. If a transcript was predicted as potential coding by at least one of these tools, it was considered as a potential protein coding sequences and excluded from the final dataset. To identify lncRNAs that act as precursors of known miRNAs, miRNA precursor sequences were downloaded from mirBase (http://www.mirbase.org/) and aligned to putative lncRNAs using BLASTn. The lncRNAs matched the miRNA precursors with 90% identification accuracy and the e-value of 1e-1 were classified as probable miRNA precursors. To determine conservation of lncRNAs, lncRNA sequences files of six plants including *Arabidopsis lyrata*, *Citrus clementinawere*, *Citrus sinensis*, *Malus domestica*, *Populus trichocarpa* and *Zea mays* were downloaded from GreeNC database (http://greenc.sciencedesigners.com/wiki/Main_Page) and then combined into a single FASTA file. These FASTA file was aligned to putative lncRNAs. The matched lncRNAs with 90% identification accuracy and the e-value of 1e-1 were classified as probable conserved lncRNAs. To prevent elimination during the next steps, the sequences of probable miRNA precursors and conserved lncRNAs were extracted from the remaining dataset. To discriminate between lncRNAs and previously annotated small noncoding RNA transcripts, we aligned all remaining transcripts against the RNAcentral database (https://rnacentral.org/). The remaining transcripts after adding probable miRNA precursors and conserved lncRNAs sequences, were introduced as candidate lncRNAs of *P. vera*, which were used for further profiling and functional analysis (Fig. 1). Comparison of some characteristics between non-coding and coding sections of transcriptome was done by QUAST tool (http://quast.bioinf.spbau.ru/).

### Detection of SSRs and transposons in lncRNAs and coding-RNAs

The MISA software (http://pgrc.ipk-gatersleben.de/misa/misa.html) was used to identify microsatellites in the reference sequences of lncRNAs and mRNAs. The SSR loci detection were done by searching of two to six nucleotides motifs with a minimum of 6,5,5,4 and 4 repeats, respectively.

To identify putative transposon sequences in lncRNAs and mRNAs, transposon sequences of six plants including *Arabidopsis thaliana* from TAIR database (https://www.arabidopsis.org/) and *Asparagus officinali*s, *Carica papaya*, *Morus notabili*s, *Populus trichocarpa* and *Phoenix dactylifera* from DPTEdb database (http://genedenovoweb.ticp.net:81/DPTEdb/index.php) were downloaded and combined into a single FASTA file. For initial filtering of transposon, reference sequences of lncRNAs and coding-RNAs were aligned to the FASTA file. The matched sequences with 90% identity and the e-value of 1e-1 were classified as probable transposon sequences. Subsequent, RepeatMasker (http://www.repeatmasker.org) was used for similarity search and repeat masking of these BLAST filtered sequences against the repeat library of *Arabidopsis thaliana*.

### Differential expression analysis

To identify differentially expressed lncRNAs under salt treatment compare to control, raw counts per transcripts were quantified using Salmon^150^ and count matrices was made for comparison of studied libraries. EdgeR package (v3.28.1)^151^ was used for analyzing of differential expression. Transcripts with a log fold change ≥ 2 and FDR < 0.01 were identified as differentially expressed lncRNAs. Integration analyses (Venn analyses) were performed. To generate a visual figure of differentially expressed lncRNAs, we generated a heat map using the R package pheatmap (v1.0.12) (https://cloud.r-project.org/web/packages/pheatmap/). Sample related to Sarakhs cultivar under 24 h salinity treatment was not sequenced due to poor RNA quality.

### Functional analysis of lncRNAs

#### Blast based transcript filtering

Natural antisense transcripts (NAT) are a specific group of ncRNA that are complementary to mRNAs. In this method, we used the following criteria to find NATs, if the paired regions between two transcripts has a continuous matching region (without gap openings) longer than 150 nucleotides with ≥ 90% identity, they were classified as 150-nt blast based filtered transcript pairs or 150-nt lnc-coding RNA-NAT pairs. For this purpose, all salt responsive lncRNAs in Gt6-Gc and Gt24-Gc were aligned against salt responsive coding genes in tolerant cultivar using BLASTn. Finally, Lnc Tar software^152^ was employed to verify the annealing potential of the BLAST predicted pairs (Supplementary Fig. S2). AgriGO web-based tool (v1.2)^153^ was used for gene ontology (GO) enrichment analysis (p < 0.05) of salt responsive coding target genes.

#### Expression pattern based transcript filtering

In this method, filtering of lncRNAs was started by drawing of Venn diagram. Venn diagram determined common differentially expressed lncRNAs among three samples of St6-Gt6, Gt6-Gc and Gt24-Gc. The target genes of these salt responsive common lncRNAs was predicted by Lnc Tar software in all salt responsive coding genes of tolerant cultivar (Supplementary Fig. S2). In order to visualize the interaction networks of these selected lncRNAs with their target genes, we used Cytoscape (v2.8)^154^. AgriGO was used for Gene ontology (GO) enrichment analysis (p < 0.05) of salt responsive coding target genes.

### Quantitative real-time PCR validation

The top five salt responsive lncRNAs with more target genes was selected for validation by qRT-PCR at three time points of 0, 6 and 24 h after salt treatment in roots of Ghazvini cultivar. To validate and compare the expression of putative pre-miRNAs and their mature miRNAs, four differential expressed miRNA-lncRNAs were selected for detecting by qRT-PCR at three time points of 0, 6 and 24 h after salt treatment in roots of Ghazvini cultivar. We used the mirBase to find mature miRNA sequences of miRNA-lncRNAs. lncRNAs and miRNA-lncRNAs primers were designed using Oligo 7 software (Supplementary Tables S6 and S7) and the actin was applied as a housekeeping gene^155^. Mature miRNA expression analysis was done by stem–loop reverse transcription-qPCR method^156,157^. Supplementary Table S8 shows the sequences of RT primers and miRNA-specific PCR primers. The 5 S rRNA was applied as a housekeeping gene.

Total RNA was isolated, from Ghazvini roots using (CTAB method). Real-time PCR was down using SYBR Green real-time PCR kit (Qiagen, Germany). The reaction mixture contained 1.0 μL of diluted cDNA sample, 0.2 μL of each of the forward and reverse primers (10 μM) and 10 μL real-time master mix with a final volume of 20 μL. The real-time PCR cycling conditions were as follows: 95 °C for 5 min for initial activation of polymerase, followed by 40 cycles at 94 °C for 30 s, 63 °C for 30 s, 72 °C for 20 s. The Pfaffl formula^158^ was used for relative gene expression calculation in REST 2009 software (http://rest.gene-quantification.info/). Three biological replicates and three technical replicates for each biological replicate were used.

## Supplementary Information

Supplementary Information.

Supplementary Information 2.
