# Supplementary material for: A genome-wide identification, characterization and functional analysis of salt-related long non-coding RNAs in non-model plant Pistacia vera L. using transcriptome high throughput sequencing
Source: Sci Rep. 2020 Mar 27;10:5585. doi: 10.1038/s41598-020-62108-6 (PMC7101358; doi:10.1038/s41598-020-62108-6)
Supplement: Supplementary file 1 — Supplementary Information. [file 41598_2020_62108_MOESM1_ESM.pdf]

A genome-wide identification, characterization and functional analysis of salt-related long non-coding RNAs in non-model plant *Pistacia vera* L. using transcriptome high throughput sequencing

Masoomeh Jannesar[1,2], Seyed Mahdi Seyedi[2]*, Maryam Moazzam Jazi[3], Vahid Niknam[1]*, Hassan Ebrahimzadeh[1] & Christopher Botanga[4]

Supplementary Information

This PDF file includes: Supplementary tables S2– S8 and supplementary figures S1 & S2

Supplementary table S2. The list of 36 conserved Pve_lncRNAs and their Blast results. Blast filtering parameters were 90 % identification accuracy and the e-value of 1e-1.

Conserved Pve_lncRNA ID	Blast result
lncRNA_PveLR3315	lcl|Alyrata_936835
lncRNA_PveLR21033	lcl|Alyrata_944213
lncRNA_PveLR10955	lcl|Cclementina_Ciclev10013256m
lncRNA_PveLR35001	lcl|Csinensis_orange1.1g047149m
lncRNA_PveLR48679	lcl|Mdomestica_MDP0000352877
lncRNA_PveLR198	lcl|Ptrichocarpa_Potri.002G182200.1
lncRNA_PveLR25592	lcl|Ptrichocarpa_Potri.009G129200.1
lncRNA_PveLR30905	lcl|Ptrichocarpa_Potri.018G127000.1
lncRNA_PveLR29137	lcl|Ptrichocarpa_Potri.010G071800.1
lncRNA_PveLR15022	lcl|Ptrichocarpa_Potri.001G242700.1
lncRNA_PveLR15021	lcl|Ptrichocarpa_Potri.001G242700.1
lncRNA_PveLR7411	lcl|Mdomestica_MDP0000336545
lncRNA_PveLR39995	lcl|Csinensis_orange1.1g034408m
lncRNA_PveLR10512	lcl|Alyrata_895156
lncRNA_PveLR34008	lcl|Cclementina_Ciclev10033161m
lncRNA_PveLR39284	lcl|Csinensis_orange1.1g034646m
lncRNA_PveLR12931	lcl|Cclementina_Ciclev10023211m
lncRNA_PveLR22668	lcl|Ptrichocarpa_Potri.015G077400.1
lncRNA_PveLR14974	lcl|Csinensis_orange1.1g035153m
lncRNA_PveLR44282	lcl|Ptrichocarpa_Potri.010G076400.1
lncRNA_PveLR9462	lcl|Csinensis_orange1.1g034788m
lncRNA_PveLR10411	lcl|Csinensis_orange1.1g035220m
lncRNA_PveLR8715	lcl|Ptrichocarpa_Potri.015G121300.1
lncRNA_PveLR34938	lcl|Csinensis_orange1.1g048653m
lncRNA_PveLR14745	lcl|Mdomestica_MDP0000174711
lncRNA_PveLR3829	lcl|Cclementina_Ciclev10024283m
lncRNA_PveLR7911	lcl|Cclementina_Ciclev10023106m
lncRNA_PveLR7172	lcl|Ptrichocarpa_Potri.014G191300.1

lncRNA_PveLR12006	lcl|Ptrichocarpa_Potri.016G029000.1
lncRNA_PveLR7226	lcl|Ptrichocarpa_Potri.003G005400.1
lncRNA_PveLR5826	lcl|Ptrichocarpa_Potri.003G046100.1
lncRNA_PveLR5825	lcl|Ptrichocarpa_Potri.003G046100.1
lncRNA_PveLR5823	lcl|Ptrichocarpa_Potri.003G046100.1
lncRNA_PveLR19634	lcl|Cclementina_Ciclev10023518m
lncRNA_PveLR17263	lcl|Csinensis_orange1.1g034943m
lncRNA_PveLR39910	lcl|Ptrichocarpa_Potri.003G046100.1

Supplementary table S3. Summary of SSR searching results.

**Item**	**Number**	
	**lncRNA**	**Coding-RNA**
Total number of sequences examined	53220	79777
Total size of examined sequences (bp)	21513694	64499690
Total number of identified SSRs	2166	4148
Number of SSR containing sequences	2051	3859
Number of sequences containing more than 1 SSR	110	254
Number of SSRs present in compound formation	44	75

Supplementary table S4. Length, GC content and pre-miRNA prediction of top-five selected lncRNA sequences.

**lncRNA name**	**Sequence length**	**GC%**	**Number of mature miRNA**	**Sequence of mature miRNA and GC%**
lncRNA_PveLR1456	528	34.28%	2	Mature miRNA 1:<br>>PveLR1456_270_22_5p cand1<br>AGUGUUUAGUAACAGCCAUAUA<br>>PveLR1456_309_22_3p cand1<br>AUUGUUGCUGAAAAUAAAUUAU<br>Mature miRNA 2:<br>>PveLR1456_333_22_5p cand2<br>UAGGUAGAUUCUUCCACCAACA<br>>PveLR1456_373_22_3p cand2<br>CGGCUGGGGCAAUCUACUGAAU
lncRNA_PveLR6140	322	33.23%	2	Mature miRNA 1:<br>>PveLR6140_0_22_5p cand1<br>GGUUUGGUCCCGGUGGUUGCAG<br>>PveLR6140_28_22_3p cand1<br>UCAGCUAAAGAGGAAUGAAUCU<br>Mature miRNA 2:<br>>PveLR6140_78_22_5p cand2<br>CUGAAAUUCUCUUGCUUAAAUU<br>>PveLR6140_142_22_3p cand2<br>UCAAGAAACAUUGAAUAAAGUU

lncRNA_PveLR6308	412	41.74%	1	Mature miRNA:<br>>PveLR6308_331_22_5p cand1<br>UGAGUGGCGGCUGAUUGAAAAA<br>>PveLR6308_366_22_3p cand1<br>AUGUAUUAUUGCUUGCAUCGGA
lncRNA_PveLR14175	620	33.06%	1	Mature miRNA:<br>>PveLR14175_503_22_5p cand1<br>ACCUUUGCUAGUAAAUCUUUCC<br>>PveLR14175_533_22_3p cand1<br>GAAUUGUUAGGUUAUUAAGGCC
lncRNA_PveLR48658	271	34.32%	1	Mature miRNA:<br>>PveLR48658_102_22_5p cand1<br>AGCGAACAACUUCCAAAGUAAA<br>>PveLR48658_152_22_3p cand1<br>GAACGUUAUUUCUAUGGGCUGC

Supplementary table S5. The list of Pve_lncRNA IDs and functional annotation of 65 annotated target genes of lnc-coding RNA-NAT pairs that were differentially expressed under salt stress predicted by Blast based transcript filtering and LncTar software.

Pve_lncRNA ID	Annotation of predicted target gene
lncRNA_PveLR40466	PYR1-like 7 (PYL7)
lncRNA_PveLR37415	AT-hook motif nuclear-localized protein 1 (AHL1)
lncRNA_PveLR45787	Plant U-box 26 (PUB26)
lncRNA_PveLR31442	GRAS family transcription factor
lncRNA_PveLR11418	Acyl-CoA N-acyltransferases (NAT) superfamily protein
lncRNA_PveLR7298	Integrase-type DNA-binding superfamily protein
lncRNA_PveLR11235	Cysteine proteinases superfamily protein
lncRNA_PveLR49741	Subtilase family protein (ATSBT3.5)
lncRNA_PveLR16776	Glutathione S-transferase tau 7 (ATGSTU7,)
lncRNA_PveLR41700	Salt tolerance zinc finger (STZ)
lncRNA_PveLR1267	RING/U-box superfamily protein
lncRNA_PveLR43855	Hydroxyproline-rich glycoprotein family protein
lncRNA_PveLR6535	Calcineurin-like metallo-phosphoesterase superfamily protein
lncRNA_PveLR32945	WRKY DNA-binding protein 33 (WRKY33)
lncRNA_PveLR11164	Gibberellin 2-oxidase 6 (ATGA2OX4)
lncRNA_PveLR6193	P-loop containing nucleoside triphosphate hydrolases superfamily protein (ISE1)
lncRNA_PveLR35325	Duplicated homeodomain-like superfamily protein
lncRNA_PveLR24929	Ethylene-responsive element binding factor 13 (ATERF13)
lncRNA_PveLR1146	Response to ABA AND SALT 1 (RAS1)
lncRNA_PveLR30510	Heat shock factor 1 (HSF1)
lncRNA_PveLR45526	UDP-glycosyltransferase (UGT)
lncRNA_PveLR36653	Sulfotransferase 17 (SOT17)
lncRNA_PveLR21263	Galacturonosyltransferase-like 10 (GATL10)
lncRNA_PveLR47667	GDSL-like Lipase/Acylhydrolase superfamily protein
lncRNA_PveLR45742	Protein kinase superfamily protein (ATN1)
lncRNA_PveLR43143	Scarecrow-like 14 (SCL14)
lncRNA_PveLR52673	Scarecrow-like 14 (SCL14)
lncRNA_PveLR26058	Scarecrow-like 14 (SCL14)
lncRNA_PveLR51611	Tetratricopeptide repeat (TPR)-containing protein (TPR14)
lncRNA_PveLR32675	Jasmonate-zim-domain protein
lncRNA_PveLR8799	RAD-like 6 (ATRL6)
lncRNA_PveLR48391	Duplicated homeodomain-like superfamily protein (RAX2)

lncRNA_PveLR47083	Sec14p-like phosphatidylinositol transfer family protein
lncRNA_PveLR5168	Integrase-type DNA-binding superfamily protein
lncRNA_PveLR26739	Heat stress transcription factor A-2c
lncRNA_PveLR21694	Concanavalin A-like lectin protein kinase family protein
lncRNA_PveLR40233	DREB and EAR motif protein 2 (DEAR2)
lncRNA_PveLR39003	Phosphotyrosine protein phosphatases superfamily protein
lncRNA_PveLR2900	RING/U-box superfamily protein (ATL3)
lncRNA_PveLR51656	Alpha/beta-Hydrolases superfamily protein
lncRNA_PveLR24975	MATE efflux family protein
lncRNA_PveLR8469	Pyruvate dehydrogenase kinase (PDK)
lncRNA_PveLR10112	Contains InterPro DOMAIN/s
lncRNA_PveLR24297	Glycolipid transfer protein (GLTP) family protein
lncRNA_PveLR9075	Tubulin/FtsZ family protein (TUA6)
lncRNA_PveLR32819	Chitin elicitor receptor kinase 1 (CERK1)
lncRNA_PveLR12882	C2 calcium/lipid-binding plant phosphoribosyltransferase family protein
lncRNA_PveLR37860	RING membrane-anchor 3 (ATRMA3)
lncRNA_PveLR53178	Late embryogenesis abundant (LEA) protein-related
lncRNA_PveLR34976	Glutamate dehydrogenase 2 (GDH2)
lncRNA_PveLR34269	Laccase 16 (LAC16)
lncRNA_PveLR25715	Ankyrin repeat family protein
lncRNA_PveLR24495	TIFY domain/Divergent CCT motif family protein (JAZ2)
lncRNA_PveLR44619	ABC transporter family protein
lncRNA_PveLR5836	INO80 ortholog (INO80)
lncRNA_PveLR37106	F-box family protein with a domain of unknown function (DUF295)
lncRNA_PveLR49041	Lysine-ketoglutarate reductase/saccharopine dehydrogenase bifunctional enzyme
lncRNA_PveLR672	Jasmonate-zim-domain protein
lncRNA_PveLR20370	Ankyrin repeat family protein
lncRNA_PveLR30912	Uridine diphosphate glycosyltransferase 74E2 (UGT74E2)
lncRNA_PveLR2014	Ethylene responsive element binding factor 1 (ATERF-1)
lncRNA_PveLR43865	Leucine-rich repeat protein kinase family protein
lncRNA_PveLR53000	Shikimate kinase 1 (SK1)
lncRNA_PveLR5534	bZIP transcription factor family protein (BZO2H3)

Supplementary table S6. Primer sequences of top five selected lncRNAs.

**lncRNA name**	**Primer sequence (5´-3´)**	**PCR product length (bp)**
lncRNA_PveLR6308	F:GTGATTGAACAGACGGGGAGTGGG<br>R:CGCTATCGCTCAGATTTGGCTTGGG	227
lncRNA_PveLR6140	F:GTCCCGGTGGTTGCAGCTAAGG<br>R:CGCATCGCGCATAACAAACTTTATTCC	174
lncRNA_PveLR1456	F:GGCGATGCTAATTTATCAAAACGTGGG<br>R:TCATATTCAGTAGATTGCCCCAGCCC	182
lncRNA_PveLR48658	F:GTTGGGAAAGATGAAGGGGATGGG<br>R:TCCAAACGCAGCCCATAGAAATAACC	129
lncRNA_PveLR14175	F:ACTATGGGATCATGTGGTGTATGTGG<br>R:GCCAACCTGAAAGTACACACAAACC	155

Supplementary table S7. Primer sequences for miRNA-lncRNAs validation.

pre-miRNA	Primer sequence (5´-3´)	PCR product length (bp)
mir319a	F: CAUCCAAAUACUGAGUUUAAGGA<br>R: AACGAUAUGUAAAAGAGUUACGG	150
mir166a	F: CTTTTGAGGGGAATGTTGTCTGGCT<br>R: TGCAAGGATGGATGAATCTGGAAGC	161
mir156g	F: GTCTATGTGTGTGGCTGGTGGTTTC<br>R: TGAGCACGCAAAAGCAAAAGTATAGC	166
mir169o	F: GGCTTCAATTCGGATCGCATTGGAA<br>R: GACTGCCTGAGAACCAAACAAAGGC	175

Supplementary table S8. Primers used in stem-loop RT-qPCR analysis

miRNA	RT primer (5´-3´)	Forward primer (5´-3´)	Reverse primer (5´-3´)
mir319a	SL37*-AGGGAGCT	FW14**-TTGGACTGAA	AACTGGTGTCGTGGAG***
mir166a	SL37-GGGGGAAT	FW14-TCGGACCAGG	
mir156g	SL37-TGTGCTCA	FW14-TGACAGAAGA	
mir169o	SL37-GCAGGCAA	FW14-TAGCCAAGGA	

SL37 = **5´**-CTCAACTGGTGTCGTGGAGTCGGCAATTCAGTTGAG-**3´***

FW14 = **5´**-ACACTCCAGCTGGG-**3´****

Identical to nt 4–19 of sequence SL37.***

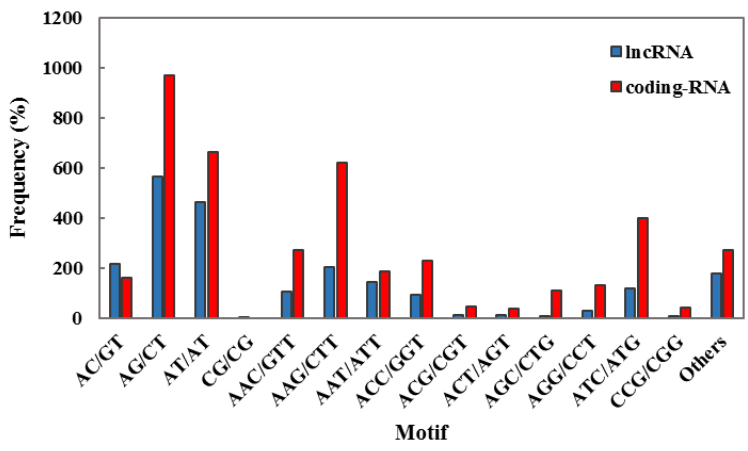

Supplementary figure S1. Frequency distribution of SSRs based on motif types in lncRNAs and coding-RNAs.

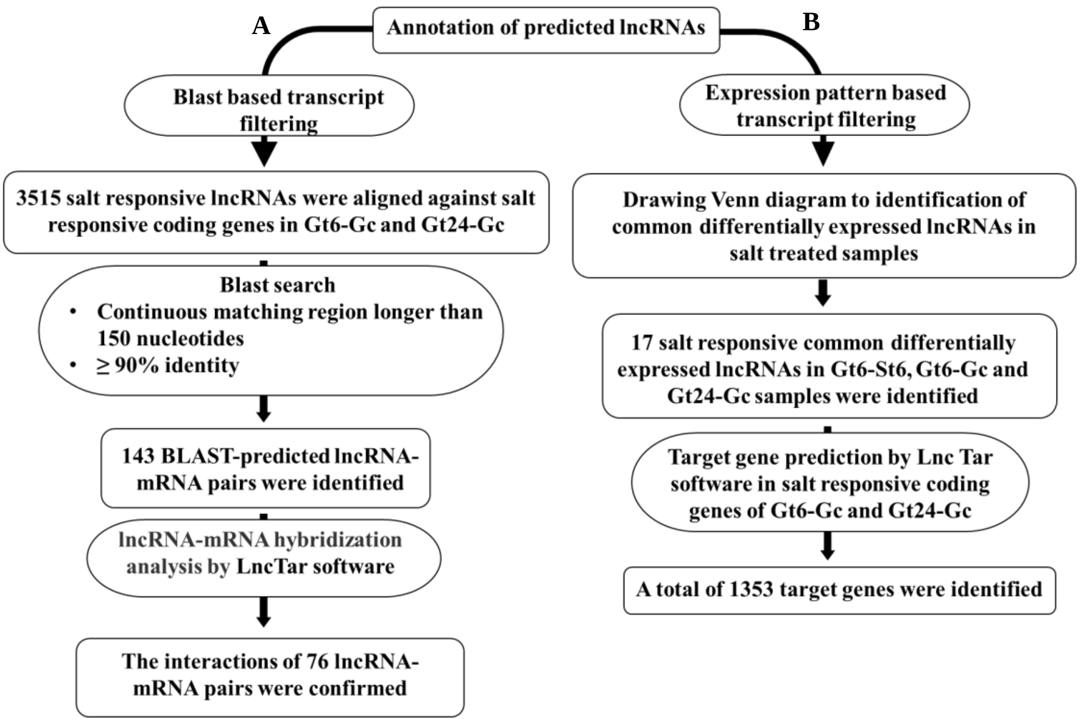

Supplementary figure S2. Summarized workflow of the pipeline used to functional analysis of lncRNAs including two methods “Blast based transcript filtering” and “Expression pattern based transcript filtering”. (A) The first method requires Blast by specific options to initial filtering of transcripts and follows with the final confirmation of the Blast predicted pairs by the Lnc Tar software. (B). In the second method, initial filtering of transcripts is done based on the expression pattern of lncRNAs. In the next step, the target genes of these selected lncRNAs were predicted by Lnc Tar software.
